# Distribution of a novel DsrEFH sulfur transferase suggests widespread sulfur oxidation capacity in sulfate reducers

**DOI:** 10.1093/ismejo/wrag130

**Published:** 2026-05-23

**Authors:** Lea Emilie Plum-Jensen, Marc Gregor Mohr, Tomohisa Sebastian Tanabe, Bo Wang, Simon Gregersen Echers, Nikoline Sanggård Madsen, Casper Thorup, Markéta Linhartová, Lars Peter Nielsen, Morten Kam Dahl Dueholm, Thomas Boesen, Ian Philip George Marshall, Christiane Dahl, Andreas Schramm

**Affiliations:** Center for Electromicrobiology, Section for Microbiology, Department of Biology, Aarhus University, DK-8000 Aarhus C,Denmark; Institute for Microbiology & Biotechnology, Rheinische Friedrich-Wilhems-Universität Bonn, D-53115 Bonn, Germany; Centre for Microbiology and Environmental Systems Science, Division of Microbial Ecology, University of Vienna, A-1030 Vienna, Austria; Center for Electromicrobiology, Section for Microbiology, Department of Biology, Aarhus University, DK-8000 Aarhus C,Denmark; Department of Chemistry and Bioscience, Aalborg University, DK-9220 Aalborg Ø, Denmark; Center for Electromicrobiology, Section for Microbiology, Department of Biology, Aarhus University, DK-8000 Aarhus C,Denmark; Department of Molecular Biology and Genetics, Aarhus University, DK-8000 Aarhus C, Denmark; Center for Electromicrobiology, Section for Microbiology, Department of Biology, Aarhus University, DK-8000 Aarhus C,Denmark; Center for Electromicrobiology, Section for Microbiology, Department of Biology, Aarhus University, DK-8000 Aarhus C,Denmark; Center for Electromicrobiology, Section for Microbiology, Department of Biology, Aarhus University, DK-8000 Aarhus C,Denmark; Department of Chemistry and Bioscience, Aalborg University, DK-9220 Aalborg Ø, Denmark; Center for Electromicrobiology, Section for Microbiology, Department of Biology, Aarhus University, DK-8000 Aarhus C,Denmark; Department of Molecular Biology and Genetics, Aarhus University, DK-8000 Aarhus C, Denmark; Center for Electromicrobiology, Section for Microbiology, Department of Biology, Aarhus University, DK-8000 Aarhus C,Denmark; Institute for Microbiology & Biotechnology, Rheinische Friedrich-Wilhems-Universität Bonn, D-53115 Bonn, Germany; Center for Electromicrobiology, Section for Microbiology, Department of Biology, Aarhus University, DK-8000 Aarhus C,Denmark

**Keywords:** sulfur metabolism, sulfur oxidation, DsrEFH, DsrAB, cable bacteria

## Abstract

Microbial sulfur cycling is typically divided into an oxidative and a reductive branch, with microbes driving either sulfide oxidation or sulfate reduction distinguished by their genomic setup. Paradoxically, filamentous cable bacteria perform electrogenic sulfide oxidation but contain genes indicative of sulfate reduction, including the reductive type of dissimilatory sulfite reductase (DsrAB), whereas they apparently lack the canonical sulfur transferase DsrEFH essential for sulfur oxidation. AlphaFold2 structure prediction of conserved cable bacteria proteins with unknown functions identified a protein complex resembling canonical DsrEFH (hereafter termed DsrEFH type II). *In vitro* characterization of heterologously expressed DsrEFH type II confirmed its sulfur transferase function and, together with site-directed mutagenesis, verified that the conserved cysteine, Cys^67^, is the active sulfur transfer residue. Genes encoding the novel DsrEFH type II were found in 985 prokaryotic genomes. They typically co-occurred with genes for reductive DsrAB in microbes characterized as sulfate reducers or sulfur disproportionators. This study not only fills an important gap in the sulfide oxidation pathway of cable bacteria, but also suggests that a wide range of sulfate reducing bacteria may be more metabolically versatile than currently understood, representing a major shift in the perception of this globally significant physiological group of microorganisms.

## Graphical abstract

**Figure:**
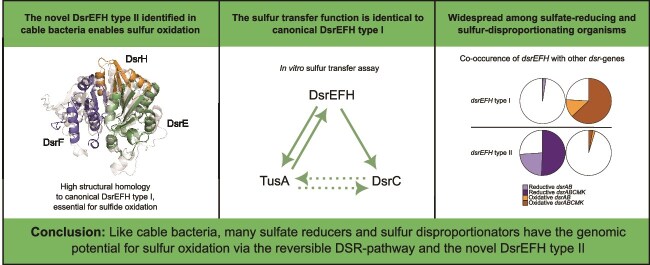

## Introduction

The anaerobic oxidation of organic carbon coupled to the respiration of sulfate is one of the most important processes in terms of global carbon turnover in the seafloor [1]. Replenishing the sulfate pool by re-oxidation of sulfide plays an equally important role [2]. Three major enzymatic systems, the dissimilatory sulfite reductase (DSR) pathway, the sulfur oxidation (SOX) pathway, and the sulfur-oxidizing heterodisulfide reductase (HDR) pathway, are involved in this process [3–5]. The DSR pathway is adaptable to function not only in the oxidative but also in the reductive direction. Thus, it occurs in both sulfate-reducing and sulfide-oxidizing prokaryotes, with dissimilatory sulfite reductase (DsrAB) as the key enzyme complex [6, 7].

**Figure 1:**
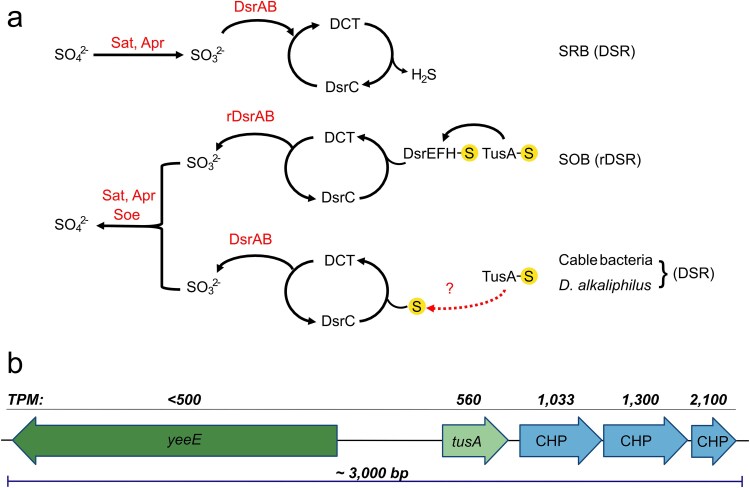
DSR-pathway in bacteria metabolizing sulfur compounds and the search for *dsrEFH* in cable bacteria. (a) DSR pathway in sulfate reducing bacteria (SRB), sulfur oxidizing bacteria (SOB), and in cable bacteria and *Desulfurivibrio alkaliphilus*. In sulfide-oxidizing organisms, reduced sulfur (highlighted) is transported across the cytoplasmic membrane and delivered by TusA to the Dsr-system. The mechanism of uptake and transport is not known in cable bacteria and *D. alkaliphilus.* Sat, sulfate adenylyl transferase; Apr, adenosine-5′-phosphosulfate reductase; Soe, sulfite-oxidizing enzyme; DCT, DsrC-trisulfide. (b) representative organization of the region within 3000 bp of the putative *dsrEFH* type II (for all cable bacteria genomes used for the pangenome analysis) and its gene expression levels as transcripts per million, TPM (for *Electronema aureum* GS). CHP, conserved hypothetical protein, in this study identified as *dsrEFH* type II.

The genes for the DsrAB complex appear phylogenetically divided into a reductive type (DsrAB) and a reverse, oxidative type (rDsrAB) [8]. They have therefore been used as genetic markers to identify the direction of the sulfur metabolic pathway in microbes (Fig. 1a), and the reductive type *dsrAB* gene abundance or expression have been quantified in environmental samples as a proxy for sulfate reduction activity [9, 10]. Other *dsr* genes have been assigned exclusively to either sulfate reduction or sulfide oxidation [11, 12], including the *dsrEFH* genes. These genes encode a sulfur transferase essential for oxidation of sulfide to sulfate [13] (Fig. 1a) and are absent in sulfate-reducing microorganisms. In brief, DSR-utilizing sulfide oxidizers are thought to contain genes for both the rDsrAB and the DsrEFH complex, whereas sulfate-reducing microorganisms are thought to encode the DsrAB complex but lack DsrEFH. Two notable exceptions to this rule have been found, where sulfur-oxidizing bacteria contain the reductive type DsrAB system and apparently lack DsrEFH. These are cable bacteria (genus *Electrothrix* and *Electronema*) [14] and *Desulfurivibrio alkaliphilus* [15, 16], both within the *Desulfobacterota* (Fig. 1a).

Cable bacteria are unique multicellular filaments that occur globally in surface sediments and conduct electrons from sulfide to oxygen over centimetre distances [17]. They can play a significant role in ecosystem health and in the cycling of sulfur [18]. Because they have not been isolated into axenic cultures, their electrogenic sulfide oxidation capacity has been derived from geochemical data and studies on enrichment cultures [17, 19]. *Desulfurivibrio alkaliphilus* is a sulfur disproportionator unable to reduce sulfate but it can oxidize sulfide with nitrate or iron oxide as electron acceptor [15, 16]. Despite their oxidative metabolism, both cable bacteria and *D. alkaliphilus* contain genes encoding enzymes of the reductive type DSR pathway (*dsrABCDMKJNRT*) along with other key genes for sulfate reduction (*aprAB, qmoABC, sat*), whereas they lack known markers for sulfide oxidation including the DsrEFH sulfur transferase. Therefore, their sulfide oxidation has been proposed to proceed via a reversed action of DsrAB [14, 15], leaving the DSR pathway partially unexplained (Fig. 1a). More recently, a so-called YTD gene cluster, encoding a YeeE/YedE-like membrane protein, the sulfur transferase TusA, and a DsrE-like protein, was identified in sulfur disproportionating organisms like *Desulfocapsa sulfexigens* [20]. Similar YTD gene clusters were also found in *D. alkaliphilus* [20] and *Electronema aureum* GS [21]; their role in either sulfur disproportionation or sulfide oxidation has remained unclear.

The goal of our study was to identify highly expressed genes with unknown functions that are conserved across the cable bacteria clade and to predict their tertiary structure. We expected that structural similarity could reveal the function of otherwise hypothetical gene products and place proteins in the central metabolism of cable bacteria that are too dissimilar in their sequences to be detected by standard protein annotation tools including hidden Markov models (HMMs) [22]. This strategy identified a putative DsrEFH complex in cable bacteria (DsrEFH type II). Using heterologous expression, we proved experimentally that DsrEFH type II had sulfur transferase activity *in vitro* similar to that of the canonical DsrEFH complex (hereafter called DsrEFH type I). Finally, we analysed the distribution and genomic context of DsrEFH type II across the microbial tree of life to assess the broader implications of our discovery for predicting the physiology of sulfur-cycling microbes.

## Methods and materials

### Genomes and pangenome analysis

Seven high-quality, single-contig genomes of cable bacteria were used for the pangenome analysis, representing diverse lineages across the cable bacteria clade. Protein-coding sequences were acquired from NCBI along with the available annotations (Supplementary Table S1). The pangenome analysis was performed on the amino acid sequences with Roary v. 3.7.0 [23] with amino acid identity cut-off = 50%, inflation value = 1.4, paralog-splitting, and core genes set to be present in all genomes.

### Transcriptome and proteome analysis

Transcriptome datasets from a single-strain enrichment of *E. aureum* GS [24] had been previously published [25, 26] (NCBI accession no. PRJNA575166). The transcriptomic reads were mapped to the high-quality genome of *E. aureum* GS [27] by bbmap v. 39.01 [28] and the average transcript per million (TPM) values of each expressed gene were calculated. Only the most highly expressed genes (>500 TPM) were included in the analysis. Expression levels of *dsrEFH* type II and their neighboring genes in the *E. aureum* GS genome were visualized with Geneious Prime v. 2022.2.2 (https://www.geneious.com).

A proteome of the same *E. aureum* GS enrichment was also publicly available [14]. Because this proteome was generated from bulk sediment and covered only approx. 10% of the *E. aureum* GS genome, three additional proteome data sets were produced for this study: (i) from whole filaments of an *Electrothrix communis* RB enrichment [29], manually collected and rinsed from sediment; (ii) from whole filaments of an *E. aureum* GS enrichment, collected in the same way; (iii) to increase proteome coverage, a sequential protein extraction protocol was established for the *E. aureum* GS enrichment, using a combination of chemical and mechanical disruption to release soluble proteins, followed by detergent treatments of the cell pellets to extract membrane-associated proteins; this procedure resulted in six protein fractions that were then analysed separately. Detailed procedures for protein extractions, sample preparation, mass spectrometry, and data analysis can be found in the Supplementary methods. The proteome data have been deposited in the MassIVE repository as MassIVE dataset MSV000099163 (*E. communis* RB), MSV000099164 (*E. aureum* GS), and MSV000099166 (fractionated proteome of *E. aureum* GS).

### Comparative omics

The core genome (Data Supplement 1) was compared to the highly expressed genes (Data Supplement 2), and genes annotated as “hypothetical proteins” within the list were identified. Three conserved hypothetical proteins (later identified as DsrEFH type II) were selected for further analysis based on their location in the genome. The tertiary protein structures of these were predicted locally with AlphaFold2 v. 2.2.4 [30]. The structures were visualized with the software PyMol v. 2.6 [31] and aligned with the “align” command with either global alignment of the structures or local alignments based on the conserved cysteine residues Cys^78^ and Cys^67^. The structural similarity to proteins of known function was analysed using the DALI-server [32]. The heterotrimer of the three proteins was predicted with AlphaFold2 and compared to a known heterotrimeric structure of DsrEFH type I (PDB ID: 2HY5) with TM-align [33]. The amino acid sequences from each type were aligned with ClustalW v. 2.1 [34] and visualized with Jalview v. 2.11.3.3 [35].

### Cloning, site-directed mutagenesis, overproduction, purification and size exclusion chromatography of recombinant proteins and sulfur transfer experiments

All bacterial strains, plasmids and primers used in this study are listed in Supplementary Table S2. The *tusA, dsrC*, and *dsrEFH* type II genes were synthesized by GenScript Biotech Corporation (New Jersey, USA). The plasmid design is outlined in the Supplementary methods.

Cysteine-to-serine exchanges were performed using the Q5 Site-Directed Mutagenesis Kit (New England Biolabs, Frankfurt, Germany) according to the manufacturer’s instructions.

Recombinant TusA, DsrC, and DsrEFH type II proteins from cable bacteria were produced in *E. coli* BL21(DE3) (Supplementary methods). Sulfur binding and transfer experiments were performed as described previously [36].

### Phylogenetic analysis of the novel DsrEFH protein sequences

The three *dsrEFH* type II genes were separately searched in the NCBI database with BLAST [37] to find organisms containing them. Hidden Markov models for the DsrEFH type II proteins were generated as previously described [38] from a dataset of 41 genomes from the BLAST results which encoded a syntenic *dsrEFH* type II gene cluster and possessed other *dsr* genes. The cutoff scores were set to a bitscore >80 for all three generated models, in accordance with the noise cutoff.

To assess the distribution of DsrEFH type II, a total of 202 601 non-redundant microbial genomes was downloaded from GlobDB version 220 [39] (https://globdb.org/home). The proteins related to sulfur metabolism in all GlobDB genomes were annotated with HMSS2 [38]. The HMM library of HMSS2 included the reductive and oxidative type DsrAB, DsrEFH type I, TusBCD, and other Dsr-proteins, and was extended by the three HMMs for DsrEFH type II proteins. If a protein had multiple above-threshold hits, the HMM with the highest bitscore was selected for the annotation.

The DsrEFH genes from each genome were concatenated into a single peptide sequence. The concatenated sequences were separately clustered for type I and type II with mcl (v. 14–137) [40]. The inflation value for the clustering method was iterated and evaluated based on distance between clusterings. Inflation values 3 and 6 were chosen for DsrEFH type I and type II respectively. For each cluster, a consensus sequence was calculated with Bio.AlignIO for Python (v. 3.12.1) [41]. The consensus sequences were then aligned with Clustal Omega (v. 2024) [42] and the amino acid sequence identity calculated between all sequences. A phylogenetic tree was calculated for the concatenated DsrEFH type II amino acid sequences with IQtree (v. 2.4.0) [43] using the maximum likelhood method and model LG + R9.

In all genomes with annotated *dsrEFH* genes, other annotated genes related to sulfur metabolism within a nucleotide distance of less than 3500 bp were considered co-localized. The limit of 3500 bp was extended for each new annotated gene in the cluster. A genome was only considered to be positive for the presence of DsrEFH types I and II if the corresponding genes were encoded in a single syntenic gene cluster. To be considered positive for the marker genes of a reductive type DSR-pathway the reductive type *dsrAB* had to be co-localized. Genomes encoding *dsrC* and syntenic *dsrMK* genes elsewhere in addition to the reductive type *dsrAB* were counted to encode the minimal gene set of the complete reductive type DSR-pathway. The same criteria were used with the genes of the oxidative rDSR-pathway.

Described isolated organisms with the *dsrEFH* type II genes were further studied by determining their phylogeny and experimentally observed physiology, and by examining the gene content of the *dsrEFH* operon. In addition, organisms that have been experimentally proven to disproportionate sulfur were analysed for their gene content. Their genomes were obtained from NCBI, annotated with Prokka [44], and used for a blastp search for the *dsrEFH* type II genes.

## Results

### Comparative multi-omics and protein structure prediction identified a novel DsrEFH complex

Seven closed or single-contig genomes from cable bacteria (Supplementary Table S1) were used to generate a high quality pangenome. The pangenome consisted of 7805 gene clusters, of which 1215 clusters (hereafter called the core genome; Data Supplement 1) were present in all seven genomes. We then compiled a list of highly expressed genes obtained from three proteomes and a transcriptome of cable bacteria. Genes were considered highly expressed when present in the proteomes or with a TPM > 500 in the transcriptome. This list included 1247 genes (Data Supplement 2). A comparison of core genes and expressed genes produced 807 genes present in both datasets, of which 48 had no annotated function. Within this subset, we identified three genes that were located adjacent to genes encoding the sulfur transporter YeeE/YedE [45, 46] and the sulfur transferase TusA [47] in all seven cable bacteria genomes (Fig. 1b). A closer inspection of the transcriptome reads from *E. aureum* GS revealed that the three genes were expressed as an operon together with *tusA* (Supplementary Fig. S1).

Polypeptide sequences derived from these three genes were used for AlphaFold2 protein structure prediction, resulting in three monomer structures (Supplementary Fig. S2a-c) of high quality based on AlphaFold2 quality assessment metrics (Supplementary Fig. S2d-f). According to the DALI database, the three proteins showed significant structural similarity to the canonical DsrEFH proteins from the phototrophic sulfur oxidizer *Allochromatium vinosum* (PDB ID: 2HY5 and 2HYB) [48]. Similarity of the monomeric protein structures to those of DsrEFH from *A. vinosum* was evaluated using several parameters: (i) The Z-score given by DALI, which indicates significantly similar structures when above 8 [32], (ii) the template modelling score (TM-score) calculated by the online TM-align tool, which indicates similarity when between 0.5–1 [33], and (iii) the root mean square deviation (RMSD) of atomic positions in Å, in which an RMSD below 3 Å often indicates homology [49]. All metrics indicate a significant structural similarity between the monomers, whereas amino acid sequence identity was always below 25% (Supplementary Table S3).

**Figure 2:**
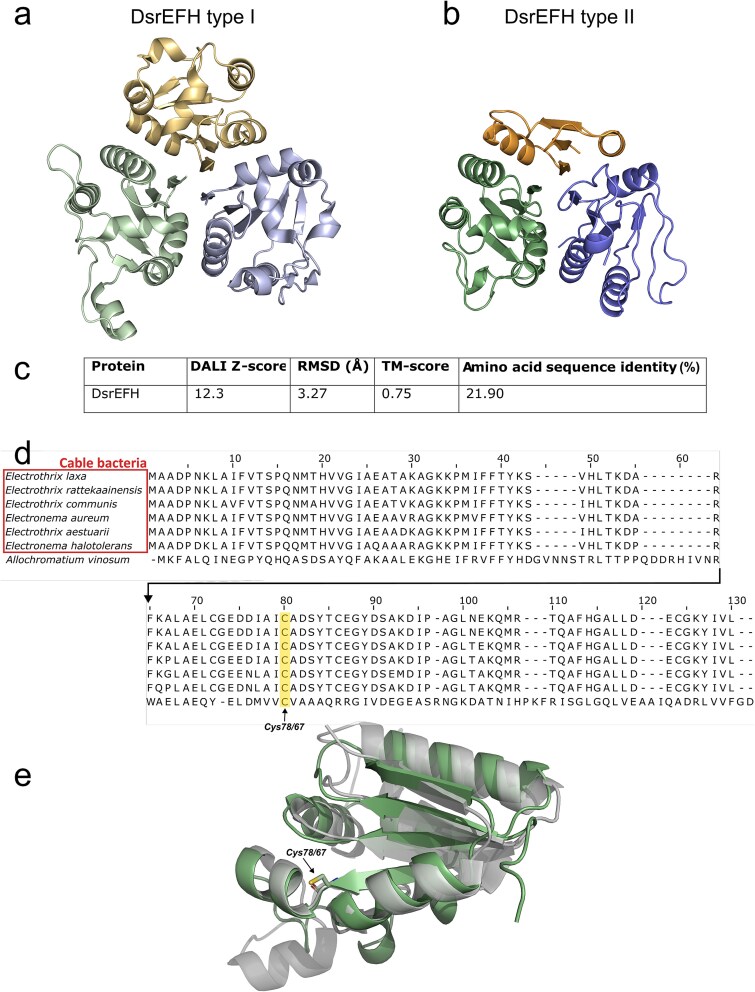
Comparison of the two heterotrimers, DsrEFH type I and DsrEFH type II. (a) Structure of the DsrEFH heterotrimer from *Allochromatium vinosum* (PDB:2HY5). Light green, DsrE; light blue, DsrF; light orange, DsrH. (b) Alphafold structural model of the putative DsrEFH type II from *Electronema aureum*. Green, DsrE; blue, DsrF; orange, DsrH. (c) Similarity metrics for the structural alignment and sequence alignment of DsrEFH type I and II. (d) Aligned amino acid sequences of DsrE type II from cable bacteria (box) and DsrE type I from *A. vinosum*. The conserved functional cysteine residue (Cys78/67) is highlighted. (e) Alignment of the DsrE subunits of the putative DsrE type II Alphafold model (green) and the structurally characterized DsrE from *A. vinosum* (PDB: 2HY5, grey). The proteins are aligned based on Cys^67^ (DsrE type II) and Cys^78^ (DsrE type I), which are visualized in stick representation. Yellow, sulfur; red, oxygen.

The significant structural resemblance of the identified proteins to the individual subunits of the well-characterized DsrEFH complex from *A. vinosum* prompted an AlphaFold2 structure prediction analysis of the putative DsrEFH proteins as a heterotrimer (Fig. 2). The *A. vinosum* enzyme crystallizes as a heterohexamer consisting of two stacked DsrEFH heterotrimers (PDB ID: 2HY5) [48]. We compared one such DsrEFH heterotrimer (Fig. 2a) and polypeptide sequence with the putative DsrEFH complex from cable bacteria (Fig. 2b), which was again predicted with high confidence according to the AlphaFold2 quality assessment metrics (Supplementary Fig. S3c, d). Although the amino acid alignment showed very low sequence identity, which can explain why this putative DsrEFH was not recognized by sequence-based annotation tools, the Z-score, TM-score, and RMSD all indicate structural homology between the two heterotrimers (Fig. 2c). Therefore, we refer to the newly identified heterotrimeric complex as DsrEFH type II, and to the canonical (*A. vinosum*) complex as DsrEFH type I (Fig. 2).

To assess the possibility that DsrEFH type II has a function similar to that of DsrEFH type I, the amino acid sequences and predicted structures were analysed for a functional sulfur transfer motif, which in DsrEFH type I is a conserved cysteine in DsrE (Cys^78^ in the *A. vinosum* polypeptide) [50]. Based on alignments of both structures and sequences of DsrEFH type I and DsrEFH type II, the functional cysteine residue was identified as the conserved Cys^67^ of DsrE type II (Fig. 2d). Aligning the structures based on these conserved cysteines resulted in an almost perfect overlay of DsrE type I and type II around the active site (Fig. 2e). The conservation of the functional motif at the structure and sequence level suggests a similar function of the two protein complexes.

### Recombinant DsrEFH type II from cable bacteria functions as sulfur transferase *in vitro*

**Figure 3:**
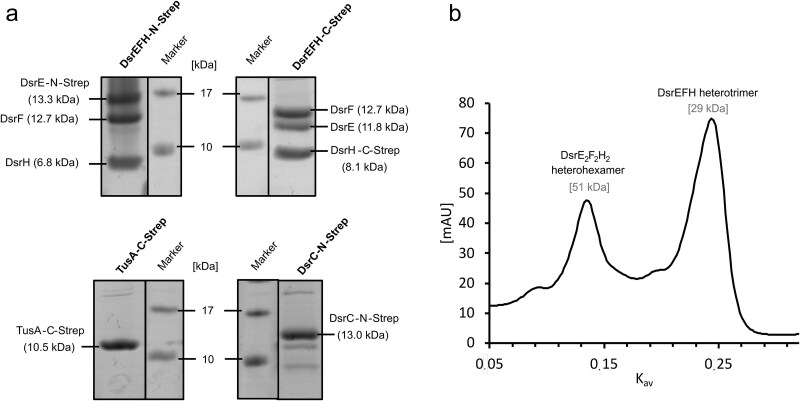
Expression in *E. coli* of recombinant genes from *Electronema aureum* GS. (a) Strep-Tactin affinity chromatography-purified recombinant DsrEFH type II, TusA, and DsrC proteins analysed by reducing SDS-PAGE. DsrE, F, and H type II from *E. aureum* GS were co-purified from *E. coli*, regardless of whether the Strep-tag was located at the N-terminus of DsrE or at the C-terminus of DsrH. Recombinant TusA carried a C-terminal Strep-tag, whereas the tag was placed at the N-terminus of DsrC. (b) Elution profile for recombinant DsrEFH type II with a C-terminal Strep-tag at DsrH upon gel filtration on Superdex 75 increase 10/300. [mAU], Milli-absorption units; K_av_, gelphase distribution coefficient*.*

To experimentally characterize the putative DsrEFH type II sulfur transferase, Strep-tagged TusA, DsrC, and DsrEFH type II from *E. aureum* GS were recombinantly produced in and purified from *E. coli* BL21(DE3) (Fig. 3a). Two versions of DsrEFH type II were made available, with the Strep-tag located either at the N-terminus of DsrE or at the C-terminus of DsrH. Regardless of the position of the Strep-tag, DsrE, DsrF, and DsrH co-eluted after affinity chromatography, indicating formation of a stable complex. Band intensities in Coomassie-stained SDS polyacrylamide gels were almost identical (Fig. 3a), indicating a 1:1:1 stoichiometry of the subunits. These conclusions were fully supported by size exclusion chromatography, where DsrEFH type II yielded two distinct peaks (Fig. 3b) with K_av_ values corresponding to 29 kDa and 51 kDa, similar to the masses predicted for DsrEFH type II heterotrimers (33 kDa) and heterohexamers (66 kDa), respectively. The heterotrimer appeared to be the predominant form.

**Figure 4:**
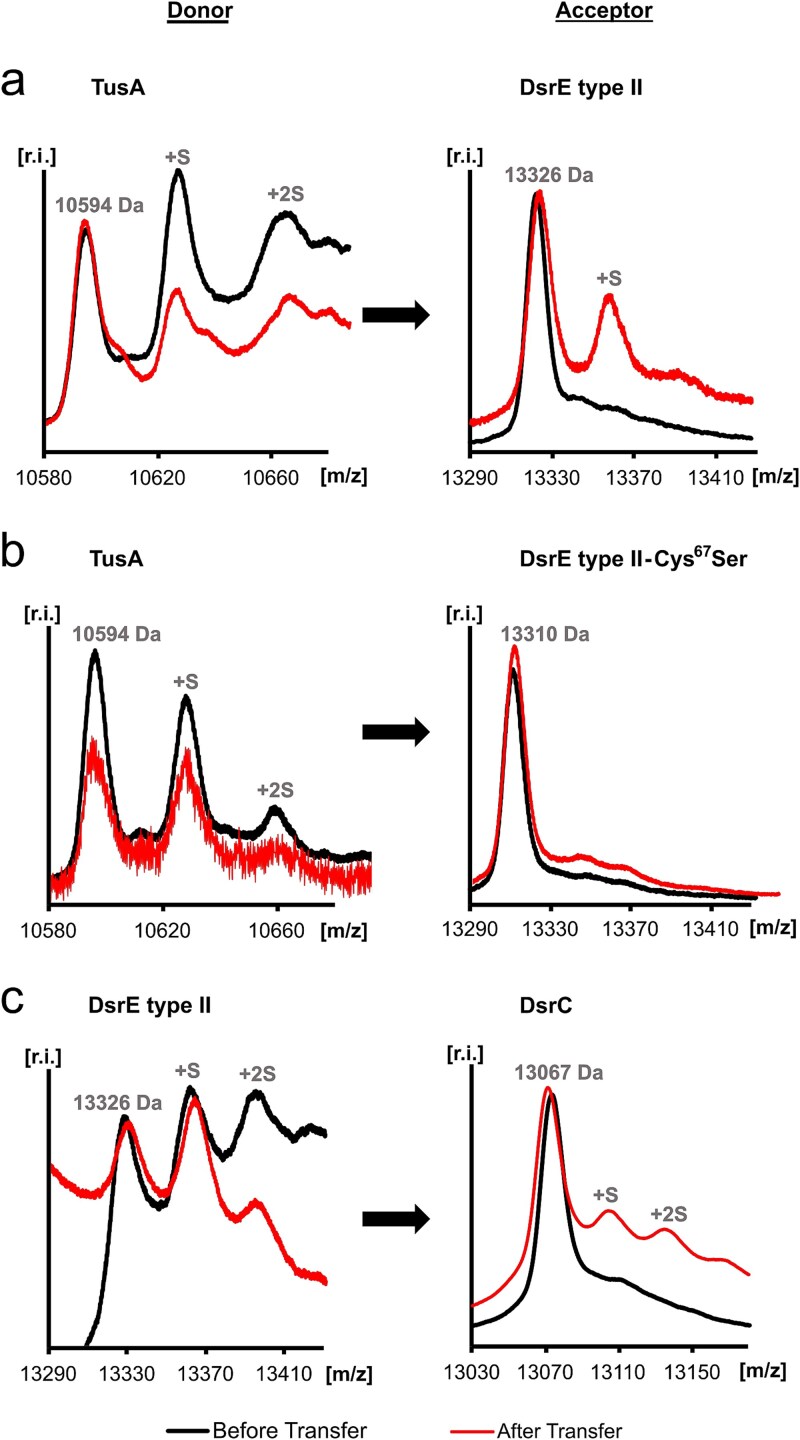
Mass spectra of sulfur transfer experiments with recombinant proteins from *Electronema aureum* GS. Left panels, donor proteins. Right panels, acceptor proteins. Spectra for donor and acceptor proteins are shown before (black line) and after (red line) sulfur transfer assay. [r.i.], relative intensity; [m/z], mass/charge ratio. (a) Sulfur transfer from TusA to DsrE type II. (b) Sulfur transfer from TusA to DsrE-Cys^67^Ser type II (exchange variant). (c) Sulfur transfer from DsrE type II to DsrC. MALDI-TOF mass spectrometry is not quantitative, and data need to be interpreted with caution. The relatively low signal intensities observed for the persulfidated DsrC species can be explained by prior transient protein–protein (here DsrEFH-DsrC) interactions [51]. Further relevant sulfur transfer assays are shown in Supplementary Figs. S4–S6. Supplementary Table S4 lists all expected masses for each protein.

The recombinant cable bacterial proteins TusA, DsrC, DsrEFH type II were used to study sulfur transfer reactions between them. The individual proteins were either persulfidated with polysulfide and used as sulfur donors, or served as sulfur acceptors in the reduced but otherwise unmodified state. Before and after the reaction, donor and acceptor proteins were analysed by MALDI-TOF mass spectrometry to assess the transfer of sulfur. The results are summarized in Supplementary Table S4. First, the sulfur transfer between TusA and DsrEFH type II was studied. Persulfidated TusA donated one sulfur atom (+32 Da) to the DsrE subunit (Fig. 4a) but did not persulfidate DsrF or DsrH (Supplementary Fig. S4a). To unambiguously identify the active sulfur-binding site in DsrE, the potential sulfur-binding cysteine, Cys^67^, was replaced by serine using site-directed mutagenesis. The cysteine-exchange variant was expressed and purified just as efficiently as the native protein and was equally stable. In contrast to native DsrE, the Cys^67^Ser variant of DsrE was no longer persulfidated by TusA (Fig. 4b, Supplementary Fig. S4b), showing that Cys^67^ indeed acts as the active sulfur-binding cysteine. After incubation of DsrEFH type II with polysulfide, DsrF and DsrE were loaded with one and up to two sulfur atoms, respectively (Supplementary Fig. S4c). Incubating proteins *in vitro* with polysulfide can lead to the nonspecific persulfidation of accessible cysteines. In addition to DsrE-Cys^67^, the cable bacterial DsrEFH type II has six cysteines, two of which are found in DsrF. One of these is in an exposed position, allowing nonspecific persulfidation. Persulfidated DsrEFH type II transferred up to two sulfur atoms to TusA (Supplementary Fig. S4c), whereas the DsrE-Cys^67^Ser variant was unable to transfer sulfur to TusA, despite being persulfidated in DsrF (Supplementary Fig. S4d). In summary, sulfur transfer *in vitro* between TusA and DsrEFH type II is bidirectional and strictly dependent on the presence of DsrE-Cys^67^.

In the next step, the transfer reactions between DsrEFH type II and DsrC were tested. Persulfidated DsrEFH type II transferred up to two sulfur atoms to DsrC (Fig. 4c, Supplementary Fig. S5a). Sulfur transfer to DsrC was also observed for the DsrE-Cys^67^Ser variant of DsrEFH type II (Supplementary Fig. S5b), most likely due to nonspecific transfer from non-active-site cysteines in DsrE and DsrF. DsrC did not function as a sulfur donor for DsrEFH type II (Supplementary Fig. S5c), showing that sulfur transfer from DsrEFH type II to DsrC is unidirectional.

In our *in vitro* assays, direct sulfur transfer between TusA to DsrC as originally proposed [14] was observed in both directions (Supplementary Fig. S6). The same has been described for the closely related TusA and DsrC proteins from *A. vinosum* [47]. Nevertheless, *in vivo* experiments have clearly identified DsrEFH type I as an essential component of the sulfur transferase cascade that feeds the rDSR-pathway in *A. vinosum* [47].

A YeeE/YedE-like putative sulfur transporter is encoded upstream of the *dsrEFH* type II genes in *E. aureum* (Fig. 1). In *E. coli*, YeeE mediates thiosulfate uptake for assimilatory sulfur acquisition and the TusA-related YeeD protein has been proposed to decompose thiosulfate, once it arrives in the cytoplasm [52]. We therefore also tested thiosulfate as persulfidation donor for the recombinant cable bacterial proteins TusA, DsrEFH type II, and DsrC. The result was negative in all cases (Supplementary Fig. S7). All cable bacteria analyzed so far lack the ability to use thiosulfate and neither contain genes for the periplasmic thiosulfate-oxidizing Sox multienzyme system nor genes encoding enzymes catalyzing formation of tetrathionate from thiosulfate (TsdA, DoxDA) [14, 27, 39, 53].

In conclusion, TusA is the only protein that is capable of transferring sulfur specifically to the active site cysteine of DsrEFH type II *in vitro*. Sulfur transfer between TusA and DsrEFH type II is bidirectional, whereas sulfur transfer between DsrEFH type II and DsrC is unidirectional *in vitro*. Our results suggest that sulfur transfer from TusA to DsrEFH type II, and from there to DsrC, likely represents the relevant reactions *in vivo*.

### Distinct sequence clusters and distributions of the two DsrEFH types across the tree of life

**Figure 5:**
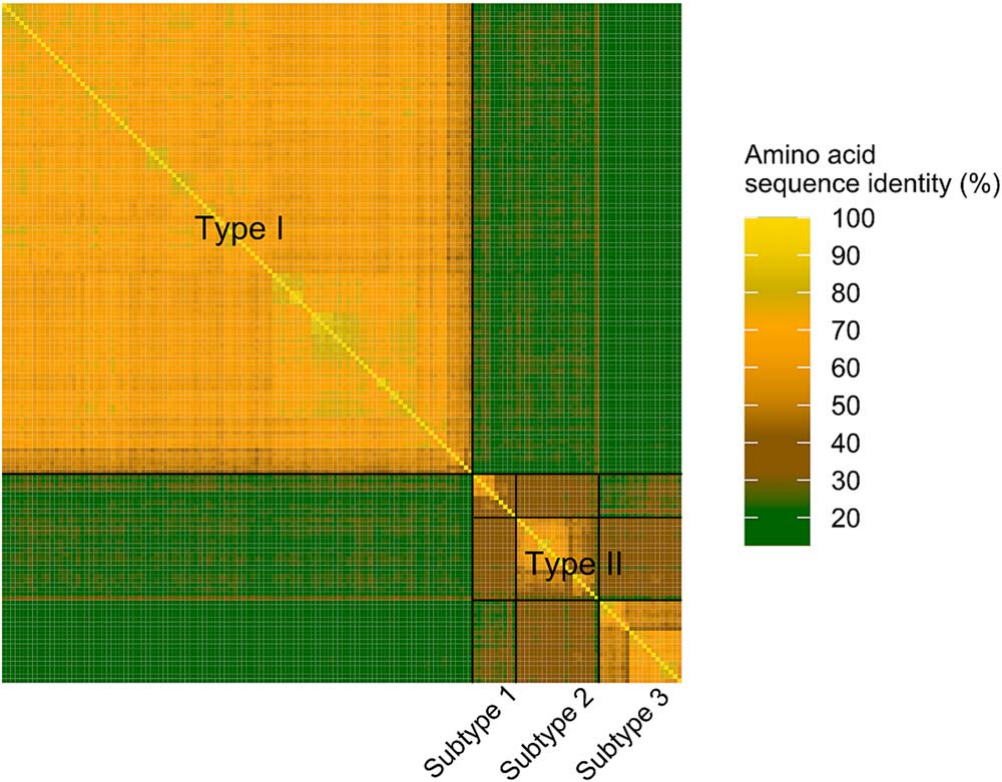
Amino acid sequence identities of DsrEFH cluster representatives of both types. Amino acid sequence identities (%) between all cluster-representative consensus sequences for DsrEFH type I and II. Yellow/orange colors in the heatmap indicate sequences with >25% identity, whereas green colors indicate sequence identities <25%. The two types form distinct clusters in the heatmap, and type II has additional subtypes based on a 40% sequence cutoff.

The 202 601 non-redundant microbial genomes of the GlobDB were searched with HMMs made from DsrEFH type II polypeptide sequences. A total of 985 genomes from 20 phyla contained all three genes in a syntenic gene cluster. The corresponding gene sequences were extracted and translated into amino acid sequences, which were concatenated and clustered. The functional cysteine residue was conserved in 99.3% of the concatenated sequences, with the remaining 0.7% scattered across the entire dataset; this random distribution suggests that the rare lack of the functional cysteine was due to sequencing errors. The search was repeated with HMMs from DsrEFH type I sequences. 3080 genomes from 18 phyla contained the genes for the DsrEFH type I complex. Amino acid sequence identities between the cluster-representative consensus sequences clearly distinguish DsrEFH type I and DsrEFH type II (shown as a heatmap in Fig. 5); only 0.3% of comparisons between DsrEFH type I consensus sequences to any of the DsrEFH type II consensus sequences had a sequence identity above 30%. The analysis also revealed that DsrEFH type I is more conserved than DsrEFH type II, and that type II can be divided into three subtypes based on a 40% amino acid sequence cutoff (Fig. 5). These subtypes are also evident in a phylogenetic analysis of all 985 DsrEFH type II sequences (Supplementary Fig. S8), where subtype 1 represents the sequences most similar to DsrEFH type I. These are found mostly in *Nitrospirota* (57%), but also in *Pseudomonadota* (21%) and *Actinomycetota* (8%), where sulfide oxidizers are more prevalent, and the genomes more often contain the oxidative type *dsrAB* (Supplementary Fig. S8). 88% of sequences from subtype 2 are found in *Desulfobacterota*, and 7% are from *Archaea*. Subtype 3 contains 99% *Desulfobacterota*.

**Figure 6:**
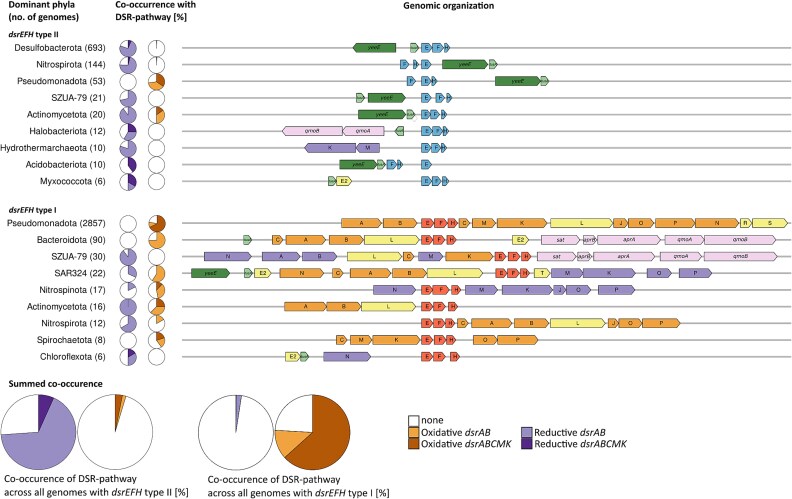
Co-occurrence of *dsrEFH* types I and II with other important genes associated with the DSR-pathway. The dominating genomic organization for phyla with more than five genomes is shown for each *dsrEFH* type. Pie charts show the percentage of co-occurrence of each *dsrEFH* type with either the reductive *dsrAB* or the oxidative *dsrAB* in purple and orange, respectively. A lighter color indicates co-occurrence with only *dsrAB*; a darker color indicates co-occurrence with *dsrABCMK* at the least. The gene clusters are labelled with either explicit gene names or letters denominating the *dsr* gene (*ABCMKLJOPNRST*). Gene color scheme: Blue, *dsrEFH* type II; red, *dsrEFH* type I; purple, reductive types of *dsr* genes; orange, oxidative types of *dsr* genes; yellow, *dsr* genes without assigned type; green/light green, *yeeE/tusA*; pink, *sat/aprAB/qmoAB*. Pie charts of the summed co-occurrences of both *dsrEFH* types with the oxidative and reductive DSR-pathways are shown at the bottom.

The GlobDB genomes encoding either of the two DsrEFH types were analysed to identify the presence of DSR-pathway marker genes and their co-occurrence and genomic organization in phyla in which more than five genomes contain the *dsrEFH* genes (Fig. 6). A minimum of *dsrAB* was used as indicator of sulfur metabolism, a minimum of *dsrABCMK* was used as indicator of a complete DSR-pathway. In addition, the *dsr*-genes were assigned to the reductive or oxidative type. The GlobDB database contains in total 3568 genomes with the reductive type *dsrAB* and 2452 genomes with the oxidative type. The majority of *dsrEFH* type I were encoded in direct vicinity to oxidative type *dsr* genes within genomes of the *Pseudomonadota* and *Bacteroidota*, which corresponds to the sulfur-oxidizing purple and green sulfur bacteria. Similar organizations were present in all other *dsrEFH* type I encoding phyla, including some with mixed type *dsr* gene clusters due to the evolutionary transition from a reductive to an oxidative type DSR-pathway [54, 55]. Overall, 75.9% of the genomes that contained *dsrEFH* type I also contained the oxidative type *dsrAB*, whereas only 2.4% contained the reductive type. In contrast, *dsrEFH* type II was primarily found in *Desulfobacterota* and *Nitrospirota* that are associated with sulfate reduction [56]. Like in cable bacteria, the bacterial *dsrEFH* type II genes were in synteny with the sulfur transferase gene *tusA* and the sulfur transporter gene *yeeE/yedE* [45, 52]. In archaea, they appeared with other genes of the sulfate reduction pathway like *dsrMK* or the genes encoding the quinol-interacting membrane-bound oxidoreductase QmoAB. Overall, 73.9% of genomes that contained *dsrEFH* type II also contained the reductive type *dsrAB*, whereas only 4.7% contained the oxidative type.

The 985 genomes that contain *dsrEFH* type II were represented by only 46 pure cultures with characterized metabolism. The majority of these (35 strains) have been described as sulfate/sulfite reducers (including 14 that were also capable of sulfur disproportionation), whereas the remaining 11 have been described solely as sulfur disproportionators (Supplementary Table S5). In five cases, the authors of the original studies explicitly stated that their isolates were tested for sulfur oxidation. Two of these, including *D. alkaliphilus,* were positive for sulfide (or polysulfide) oxidation, and two were positive for oxidation of either S^0^, thiosulfate, or sulfite, but not sulfide. To investigate whether *dsrEFH* type II is generally found in sulfur disproportionators, we performed a literature search for known sulfur disproportionating microorganisms and retrieved another 38 isolates in addition to the 25 strains with *dsrEFH* type II mentioned above (Supplementary Table S6). This means that of 63 known sulfur disproportionators available in pure culture, only 25 carry *dsrEFH* type II (40%). Finally, we tested, if *dsrEFH* type II was associated with a specific disproportionation pathway, namely the disproportionation of either sulfite, elemental sulfur, or thiosulfate (Supplementary Fig. S9); this was not the case (Supplementary Fig. S9d; Supplementary Table S6).

## Discussion

### Sulfur oxidation in cable bacteria

Biogeochemical evidence implies that cable bacteria oxidize sulfide in anoxic sediment and conduct the resulting electrons through periplasmic fibers to the oxic sediment surface where they perform oxygen reduction [17, 19]. However, the absence of pure cultures [24, 29, 57] has prevented the definite proof of this metabolism, and the genomic prediction of sulfide oxidation has remained rather inconclusive due to the presence of a reductive type DsrAB and the absence of the DsrEFH diagnostic marker for sulfide oxidation [14, 58]. Here we show that cable bacteria do encode a functional but alternative type of the DsrEFH complex. This DsrEFH type II was not identified using currently available sequence-based annotation tools and has very low amino acid sequence identity (<25%) to DsrEFH type I from known sulfide oxidizers like *A. vinosum*. Rather, the similarity between the two types is apparent from comparison of their protein structures and from the identification of a single conserved, functional motif (Fig. 2). Other *dsr* genes, e.g. *dsrJNT*, had also remained unannotated in cable bacteria, but with the advances in genomics and structural protein predictions, these “missing” proteins have recently also been identified [53, 57].

**Figure 7:**
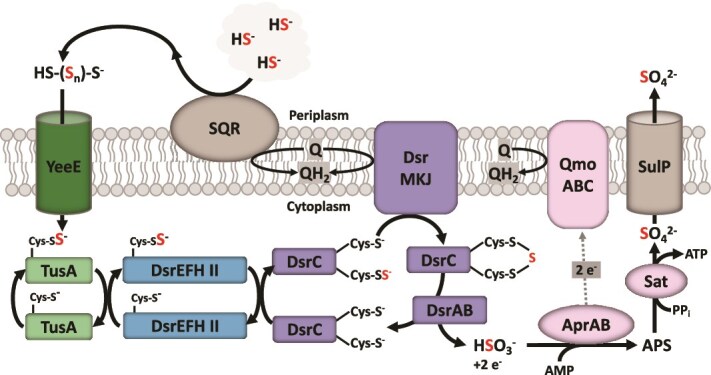
Proposed pathway for sulfide oxidation in the cable bacterium *Electronema aureum* GS. Sulfide (HS^−^) is oxidized by a type III sulfide:quinone oxidoreductase (SQR; locus tag LDFHOB_09195). The active site of this monotopic membrane protein is thought to be oriented towards the periplasm and electrons are transferred to the quinone pool. Polysulfides (^−^S–(S_n_)–SH) are formed and are likely substrates for sulfane sulfur translocation across the membrane via the YeeE-like transporter (LDFHOB_04565). In the cytoplasm, sulfane sulfur is accepted by TusA (LDFHOB 04575) and subsequently transferred via DsrEFH type II (LDFHOB_04580–04590) to DsrC (LDFHOB_03635). Formation of a DsrC trisulfide and quinone reduction is catalyzed by the membrane-bound DsrMKJ complex (LDFHOB_13320–13 330/LDFHOB_01150/01145). Reductive-type DsrAB (LDFHOB_06755/ 06760) oxidizes the central sulfur in the DsrC-trisulfide to sulfite, which is further oxidized to the sulfonate state in APS by AprAB (LDFHOB_02330/02325). The released electrons are transferred to the quinone pool by the membrane-bound QmoABC complex (LDFHOB_02335–02345). Finally, sulfate adenylyltransferase, Sat (LDFHOB_10140) catalyzes formation of ATP and sulfate, which is exported by SulP (LDFHOB_01330/03320).

The genes encoding DsrEFH type II in cable bacteria are part of an operon that also encodes the sulfur transferase TusA and are located adjacent to the gene for the YeeE/YedE-like sulfur transporter (Fig. 1b). This genomic region corresponds to the previously described YTD cluster, which comprises a *yedE*-related gene, a *tusA* gene, a *dsrE*-like gene, and two genes encoding conserved hypothetical proteins (CHPs) [20]; here we identify the *dsrE*-like gene and the two CHPs as *dsrEFH* type II. The YTD cluster appears upregulated during thiosulfate disproportionation in *Desulfolithobacter dissulfuricans* [59] and has been discussed as molecular marker for sulfur disproportionation [20, 21]; in contrast, our *in vitro* analysis of DsrEFH type II (Fig. 4) strongly suggests that it is part of a DSR-based sulfur oxidation pathway: *Electronema aureum GS* possesses a type III sulfide:quinone oxidoreductase with its active site presumably facing the periplasm [14, 60, 61]. Polysulfides are the products of the SQR reaction and electrons are transferred to the quinone pool (Fig. 7). The polysulfides might be translocated across the membrane by the cable bacterial YeeE-like protein, similar to sulfane sulfur translocation via the YeeE-family transporter SoxT1 in *Hyphomicrobium denitrificans* [45]. TusA would serve as an acceptor for the sulfur, once it arrives in the cytoplasm. Analogous to the sulfur transfer cascade in *A. vinosum* [47, 50], TusA-bound sulfur is then moved to DsrEFH type II, relying on its specific cysteine residue Cys^67^, followed by transfer to DsrC. In a reaction reverse to that described for sulfate reducers, the persulfidated DsrC is then oxidized to DsrC-trisulfide [62, 63], which serves as substrate for DsrAB, finally resulting in the oxidation of sulfur to sulfite. The key difference is that *A. vinosum* uses the oxidative type DsrAB, whereas cable bacteria use the reductive type DsrAB in reverse (Fig. 1a, Fig. 7). Finally, adenosine-5′-phosphosulfate (APS) reductase (AprAB) and sulfate adenylyltransferase (Sat) oxidize sulfite to sulfate. This model does not exclude that the YTD cluster (incl. DsrEFH type II) and the reductive type DsrAB are involved in the oxidative part of S^0^ disproportionation, specifically the oxidation of elemental sulfur to sulfite (Supplementary Fig. S9), as indicated for *Desulfurivibrio* species [24, 64]). Our model is, however, very different from a recent proposal (based on comparative genomics alone) that sulfide in cable bacteria is first oxidized to sulfite, which is then disproportionated to sulfide and sulfate [53].

### DsrEFH diversity and distribution in sulfur-cycling prokaryotes

The novel *dsrEFH* type II gene cluster is not restricted to cable bacteria but, as already indicated by the wider occurrence of the YTD gene cluster [20, 21, 65], can be found in 985 genomes from 20 phyla across the prokaryotic tree of life (Fig. 6, Fig. S8). Compared to DsrEFH type I, there is more sequence diversity in DsrEFH type II and a division into three, phylogentically distinct subclusters (Fig. 5); amino acid sequence identities to DsrEFH type I remain in all cases below 30% (Fig. 5) making it difficult to infer a common ancestry for the two DsrEFH types [66]. However, several factors indicate significant homology, including the conserved cysteine residue, the conserved structure of each individual monomer, and the DsrEFH type II phylogeny (Fig. S8), which may suggest a transition from DsrEFH type I to type II within the *Nitrospirota* or *Pseudomonadota*.

In terms of gene content, *dsrEFH* type I typically co-occurs with oxidative *dsrAB*, whereas *dsrEFH* type II co-occurs with the reductive type *dsrAB* (Fig. 6). Given the *in silico* and *in vitro* analysis of the conserved structure and function of DsrEFH type II, and its consistent co-occurrence with *yeeE/yedE* and *tusA,* it is conceivable that DsrEFH type II generally allows microbes with the reductive type DsrAB to reverse their DSR-pathway to oxidize sulfur, as described above for cable bacteria. To date, beyond cable bacteria, only five strains with this genomic setup have been tested for sulfur oxidation, four of them positively (Supplementary Table S5); still, an oxidative capacity of DsrAB remains to be proven at the enzyme level. We propose that DsrEFH type II indicates a general capacity to oxidize sulfur, where the specificity for distinct sulfur compounds (e.g. sulfide, thiosulfate, or polysulfide) is likely conferred by the sulfur uptake and transport system. The alternative hypothesis, that the *yeeE/yedE*-*tusA-dsrEFH* type II (formerly YTD) gene cluster is essential for sulfur disproportionation [20, 59], is poorly supported, because there are sulfur-disproportionating prokaryotes both with and without *dsrEFH* type II (Supplementary Tables S5, S6), and the presence of *dsrEFH* type II in sulfur-disproportionating organisms does not correlate with the ability to disproportionate specific sulfur species, such as elemental sulfur, either (see Supplementary Discussion, Table S6, Fig. S9). In addition, many sulfate reducers incapable of disproportionation contain *dsrEFH* type II (Supplementary Table S5). A recent comparative genomics study of sulfur disproportionators [65] also came to the conclusion that there must be multiple pathways for sulfur disproportionation depending on the taxa and substrates involved. Therefore, although it is still conceivable that *dsrEFH* type II is involved in the oxidation reaction of specific sulfur disproportionators (see [65] and Supplementary discussion), the most likely interpretation is that it presents a new marker for sulfur oxidation capacity in sulfate-reducing and sulfur-disproportionating prokaryotes. If so, ~20% of the known sulfate reducers and sulfur disproportionators with the reductive type DsrAB should be able to oxidize sulfur compounds. This hypothesis should be experimentally tested, and if proven correct, will change the understanding of sulfur cycling communities and the prediction of their biogeochemical impact.

## Supplementary Material

Supplementary_material_wrag130

## Data Availability

The datasets generated and analysed during the current study are available from the MassIVE repository (datasets MSV000099163, MSV000099164, and MSV000099166) and NCBI (accession no. PRJNA575166), respectively.

## References

[1] Jørgensen BB, Egger M, Canfield DE. Sulfate distribution and sulfate reduction in global marine sediments. *Geochim Cosmochim Acta* 2024;364:79–88. 10.1016/j.gca.2023.11.015

[2] Thullner M, Dale AW, Regnier P. Global-scale quantification of mineralization pathways in marine sediments: a reaction-transport modeling approach. *Geochem Geophys Geosyst* 2009;10:10. 10.1029/2009GC002484

[3] Pott AS, Dahl C. Sirohaem sulfite reductase and other proteins encoded by genes at the *dsr* locus of *Chromatium vinosum* are involved in the oxidation of intracellular sulfur. *Microbiology* 1998;144:1881–94. 10.1099/00221287-144-7-1881 9695921

[4] Friedrich CG, Quentmeier A, Bardischewsky F et al. Novel genes coding for lithotrophic sulfur oxidation of *Paracoccus pantotrophus* GB17. *J Bacteriol* 2000;182:4677–87. 10.1128/JB.182.17.4677-4687.2000 10940005 PMC111341

[5] Koch T, Dahl C. A novel bacterial sulfur oxidation pathway provides a new link between the cycles of organic and inorganic sulfur compounds. *ISME J* 2018;12:2479–91. 10.1038/s41396-018-0209-7 29930335 PMC6155103

[6] Oliveira TF, Vonrhein C, Matias PM et al. The crystal structure of *Desulfovibrio vulgaris* dissimilatory sulfite reductase bound to DsrC provides novel insights into the mechanism of sulfate respiration. *J Biol Chem* 2008;283:34141–9. 10.1074/jbc.M805643200 18829451 PMC2662231

[7] Dahl C, Engels S, Pott-Sperling AS et al. Novel genes of the *dsr* gene cluster and evidence for close interaction of Dsr proteins during sulfur oxidation in the phototrophic sulfur bacterium *Allochromatium vinosum*. *J Bacteriol* 2005;187:1392–404. 10.1128/jb.187.4.1392-1404.2005 15687204 PMC545617

[8] Müller AL, Kjeldsen KU, Rattei T et al. Phylogenetic and environmental diversity of DsrAB-type dissimilatory (bi)sulfite reductases. *ISME J* 2015;9:1152–65. 10.1038/ismej.2014.208 25343514 PMC4351914

[9] Leloup J, Petit F, Boust D et al. Dynamics of sulfate-reducing microorganisms (*dsrAB* genes) in two contrasting mudflats of the Seine estuary (France). *Microb Ecol* 2005;50:307–14. 10.1007/s00248-004-0034-6 16308673

[10] Rodriguez-Mora MJ, Edgcomb VP, Taylor C et al. The diversity of sulfide oxidation and sulfate reduction genes expressed by the bacterial communities of the Cariaco Basin. *Venezuela Open Microbiol J* 2016;10:140–9. 10.2174/1874285801610010140 27651847 PMC5012083

[11] Löffler M, Wallerang KB, Venceslau SS et al. The iron-sulfur flavoprotein DsrL as NAD(P)H:acceptor oxidoreductase in oxidative and reductive dissimilatory sulfur metabolism. *Front Microbiol* 2020;11:578209. 10.3389/fmicb.2020.578209 33178160 PMC7596348

[12] Ferreira D, Barbosa ACC, Oliveira GP et al. The DsrD functional marker protein is an allosteric activator of the DsrAB dissimilatory sulfite reductase. *PNAS* 2022;119:e2118880119. 10.1073/pnas.2118880119 35064091 PMC8794893

[13] Cort JR, Selan U, Schulte A et al. *Allochromatium vinosum* DsrC: solution-state NMR structure, redox properties, and interaction with DsrEFH, a protein essential for purple sulfur bacterial sulfur oxidation. *J Mol Biol* 2008;382:692–707. 10.1016/j.jmb.2008.07.022 18656485 PMC2637153

[14] Kjeldsen KU, Schreiber L, Thorup CA et al. On the evolution and physiology of cable bacteria. *PNAS* 2019;116:19116–25. 10.1073/pnas.1903514116 31427514 PMC6754541

[15] Thorup C, Schramm A, Findlay AJ et al. Disguised as a sulfate reducer: growth of the deltaproteobacterium *Desulfurivibrio alkaliphilus* by sulfide oxidation with nitrate. *mBio* 2017;8:e00671–17. 10.1128/mBio.00671-17 28720728 PMC5516251

[16] Chen S-C, Li X-M, Battisti N et al. Microbial iron oxide respiration coupled to sulfide oxidation. *Nature* 2025;646:925–33. 10.1038/s41586-025-09467-0 40866705 PMC12545173

[17] Pfeffer C, Larsen S, Song J et al. Filamentous bacteria transport electrons over centimetre distances. *Nature* 2012;491:218–21. 10.1038/nature11586 23103872

[18] Seitaj D, Schauer R, Sulu-Gambari F et al. Cable bacteria generate a firewall against euxinia in seasonally hypoxic basins. *PNAS* 2015;112:13278–83. 10.1073/pnas.1510152112 26446670 PMC4629370

[19] Risgaard-Petersen N, Revil A, Meister P et al. Sulfur, iron-, and calcium cycling associated with natural electric currents running through marine sediment. *Geochim Cosmochim Acta* 2012;92:1–13. 10.1016/j.gca.2012.05.036

[20] Umezawa K, Kojima H, Kato Y et al. Disproportionation of inorganic sulfur compounds by a novel autotrophic bacterium belonging to *Nitrospirota*. *Syst Appl Microbiol* 2020;43:126110. 10.1016/j.syapm.2020.126110 32847785

[21] Barbosa ACC, Venceslau SS, Ferreira D et al. Characterization of DsrD and its interaction with the DsrAB dissimilatory sulfite reductase. *Protein Sci* 2024;33:e5222. 10.1002/pro.5222 39548845 PMC11568415

[22] Whisstock JC, Lesk AM. Prediction of protein function from protein sequence and structure. *Q Rev Biophys* 2003;36:307–40. 10.1017/S0033583503003901 15029827

[23] Page AJ, Cummins CA, Hunt M et al. Roary: rapid large-scale prokaryote pan genome analysis. *Bioinformatics* 2015;31:3691–3. 10.1093/bioinformatics/btv421 26198102 PMC4817141

[24] Thorup C, Petro C, Bøggild A et al. How to grow your cable bacteria: establishment of a stable single-strain culture in sediment and proposal of *Candidatus* Electronema aureum GS. *Syst Appl Microbiol* 2021;44:126236–6. 10.1016/j.syapm.2021.126236 34332367

[25] Marzocchi U, Thorup C, Dam AS et al. Dissimilatory nitrate reduction by a freshwater cable bacterium. *ISME J* 2022;16:50–7. 10.1038/s41396-021-01048-z 34215856 PMC8692496

[26] Thorup C . Omics Insight into Cable Bacteria Metabolism. PhD thesis. Aarhus, Denmark: Aarhus University, 2020.

[27] Sereika M, Petriglieri F, Jensen TBN et al. Closed genomes uncover a saltwater species of *Candidatus* Electronema and shed new light on the boundary between marine and freshwater cable bacteria. *ISME J* 2023;17:561–9. 10.1038/s41396-023-01372-6 36697964 PMC10030654

[28] Bushnell B . BBMap: A Fast, Accurate, Splice-Aware Aligner. Berkeley, CA, USA: Lawrence Berkeley National Laboratory, LBNL-7065E, 2014, Available: https://escholarship.org/uc/item/1h3515gn.

[29] Plum-Jensen LE, Schramm A, Marshall IPG. First single-strain enrichments of *Electrothrix* cable bacteria, description of *E. Aestuarii* sp. nov. and *E. Rattekaaiensis* sp. nov., and proposal of a cable bacteria taxonomy following the rules of the SeqCode. *Syst Appl Microbiol* 2024;47:126487. 10.1016/j.syapm.2024.126487 38295603

[30] Jumper J, Evans R, Pritzel A et al. Highly accurate protein structure prediction with AlphaFold. *Nature* 2021;596:583–9. 10.1038/s41586-021-03819-2 34265844 PMC8371605

[31] DeLano WL. The PyMOL Molecular Graphics System, Version 2.6, Schrödinger LLC, New York, NY, USA 2025, Available: https://www.pymol.org.

[32] Holm L . Dali server: structural unification of protein families. *Nucleic Acids Res* 2022;50:W210–5. 10.1093/nar/gkac387 35610055 PMC9252788

[33] Zhang Y, Skolnick J. TM-align: a protein structure alignment algorithm based on the TM-score. *Nucleic Acids Res* 2005;33:2302–9. 10.1093/nar/gki524 15849316 PMC1084323

[34] Thompson JD, Gibson TJ, Higgins DG. Multiple sequence alignment using ClustalW and ClustalX. *Curr Prot Bioinform* 2003;00:2.3.1–22. 10.1002/0471250953.bi0203s00 18792934

[35] Waterhouse AM, Procter JB, Martin DMA et al. Jalview version 2—a multiple sequence alignment editor and analysis workbench. *Bioinformatics* 2009;25:1189–91. 10.1093/bioinformatics/btp033 19151095 PMC2672624

[36] Tanabe TS, Bach E, D'Ermo G et al. A cascade of sulfur transferases delivers sulfur to the sulfur-oxidizing heterodisulfide reductase-like complex. *Protein Sci* 2024;33:e5014. 10.1002/pro.5014 38747384 PMC11094781

[37] Sayers EW, Bolton EE, Brister JR et al. Database resources of the national center for biotechnology information. *Nucleic Acids Res* 2022;50:D20–6. 10.1093/nar/gkab1112 34850941 PMC8728269

[38] Tanabe TS, Dahl C. HMS-S-S: a tool for the identification of sulphur metabolism-related genes and analysis of operon structures in genome and metagenome assemblies. *Mol Ecol Resources* 2022;22:2758–74. 10.1111/1755-0998.13642 35579058

[39] Speth DR, Pullen N, Aroney STN et al. GlobDB: a comprehensive species-dereplicated microbial genome resource. *Bioinf Adv* 2025;5:vbaf280. 10.1093/bioadv/vbaf280 PMC12629238 41268474

[40] van Dongen S . Graph clustering via a discrete uncoupling process. *Siam J Matrix Analysis Appl* 2008;30:121–41. 10.1137/040608635

[41] Cock PA, Antao T, Chang JT et al. Biopython: freely available python tools for computational molecular biology and bioinformatics. *Bioinformatics* 2009;25:1422–3. 10.1093/bioinformatics/btp163 19304878 PMC2682512

[42] Madeira F, Madhusoodanan N, Lee J et al. The EMBL-EBI job dispatcher sequence analysis tools framework in 2024. *Nucleic Acids Res* 2024;52:W521–5. 10.1093/nar/gkae241 38597606 PMC11223882

[43] Minh Q, Schmidt HA, Chernomor O et al. IQ-TREE 2: new models and efficient methods for phylogenetic inference in the genomic era. *Mol Biol Evol* 2020;37:1530–4. 10.1093/molbev/msaa015 32011700 PMC7182206

[44] Seemann T . Prokka: rapid prokaryotic genome annotation. *Bioinformatics* 2014;30:2068–9. 10.1093/bioinformatics/btu153 24642063

[45] Li J, Göbel F, Hsu HY et al. YeeE-like bacterial SoxT proteins mediate sulfur import for oxidation and signal transduction. *Commun Biol* 2024;7:1548. 10.1038/s42003-024-07270-7 39572704 PMC11582611

[46] Tanaka Y, Yoshikaie K, Takeuchi A et al. Crystal structure of a YeeE/YedE family protein engaged in thiosulfate uptake. *Sci Adv* 2020;6:eaba7637. 10.1126/sciadv.aba7637 32923628 PMC7449682

[47] Stockdreher Y, Sturm M, Josten M et al. New proteins involved in sulfur trafficking in the cytoplasm of *Allochromatium vinosum*. *J Biol Chem* 2014;289:12390–403. 10.1074/jbc.M113.536425 24648525 PMC4007435

[48] Dahl C, Schulte A, Shin DH. Cloning, expression, purification, crystallization and preliminary X-ray diffraction analysis of DsrEFH from *Allochromatium vinosum*. *Acta Crystallogr Sect F: Struct Biol Cryst Commun* 2007;63:890–2. 10.1107/S1744309107041188 PMC2339738 17909298

[49] Reva BA, Finkelstein AV, Skolnick J. What is the probability of a chance prediction of a protein structure with an RMSD of 6 å? *Fold Des* 1998;3:141–7. 10.1016/S1359-0278(98)00019-4 9565758

[50] Dahl C, Schulte A, Stockdreher Y et al. Structural and molecular genetic insight into a widespread sulfur oxidation pathway. *J Mol Biol* 2008;384:1287–300. 10.1016/j.jmb.2008.10.016 18952098

[51] Yanes O, Villanueva J, Querol E et al. Detection of non-covalent protein interactions by 'intensity fading' MALDI-TOF mass spectrometry: applications to proteases and protease inhibitors. *Nat Protoc* 2007;2:119–30. 10.1038/nprot.2006.487 17401346

[52] Ikei M, Miyazaki R, Monden K et al. YeeD is an essential partner for YeeE-mediated thiosulfate uptake in bacteria and regulates thiosulfate ion decomposition. *PLoS Biol* 2024;22:e3002601. 10.1371/journal.pbio.3002601 38656967 PMC11073785

[53] Hiralal A, Geelhoed JS, Neukirchen S et al. The genetic repertoire underlying electrogenic sulphur oxidation in cable bacteria. *BMC Genomics* 2026;27:340. 10.1186/s12864-026-12675-1 41814157 PMC13047784

[54] Neukirchen S, Pereira IAC, Sousa FL. Stepwise pathway for early evolutionary assembly of dissimilatory sulfite and sulfate reduction. *ISME J* 2023;17:1680–92. 10.1038/s41396-023-01477-y 37468676 PMC10504309

[55] Klier KM, Martin C, Langwig MV et al. Evolutionary history and origins of Dsr-mediated sulfur oxidation. *ISME J* 2024;18:wrae167. 10.1093/ismejo/wrae167 39206688 PMC11406059

[56] Diao M, Dyksma S, Koeksoy E et al. Global diversity and inferred ecophysiology of microorganisms with the potential for dissimilatory sulfate/sulfite reduction. *FEMS Microbiol Reviews* 2023;47:fuad058. 10.1093/femsre/fuad058 PMC10591310 37796897

[57] Hiralal A, Geelhoed JS, Hidalgo-Martinez S et al. Closing the genome of unculturable cable bacteria using a combined metagenomic assembly of long and short sequencing reads. *Microb Genom* 2024;10:001197. 10.1099/mgen.0.001197 38376381 PMC10926707

[58] Anantharaman K, Hausmann B, Jungbluth SP et al. Expanded diversity of microbial groups that shape the dissimilatory sulfur cycle. *ISME J* 2018;12:1715–28. 10.1038/s41396-018-0078-0 29467397 PMC6018805

[59] Hashimoto Y, Shimamura S, Tame A et al. Physiological and comparative proteomic characterization of *Desulfolithobacter dissulfuricans* gen. nov., sp. nov., a novel mesophilic, sulfur-disproportionating chemolithoautotroph from a deep-sea hydrothermal vent. *Front Microbiol* 2022;13:13. 10.3389/fmicb.2022.1042116 PMC9751629 36532468

[60] Cherney MM, Zhang Y, Solomonson M et al. Crystal structure of sulfide:quinone oxidoreductase from *Acidithiobacillus ferrooxidans*: insights into sulfidotrophic respiration and detoxification. *J Mol Biol* 2010;398:292–305. 10.1016/j.jmb.2010.03.018 20303979

[61] Zhang Y, Weiner JH. Characterization of the kinetics and electron paramagnetic resonance spectroscopic properties of *Acidithiobacillus ferrooxidans* sulfide:quinone oxidoreductase (SQR). *Arch Biochem Biophys* 2014;564:110–9. 10.1016/j.abb.2014.09.016 25303790

[62] Barbosa ACC, Venceslau SS, Pereira IAC. DsrMKJOP is the terminal reductase complex in anaerobic sulfate respiration. *PNAS* 2024;121:e2313650121. 10.1073/pnas.2313650121 38285932 PMC10861901

[63] Kümpel C, Grosser M, Tanabe TS et al. Fe/S proteins in microbial sulfur oxidation. *Biochim Biophys Acta (BBA) - Mol Cell Res* 2024;1871:119732. 10.1016/j.bbamcr.2024.119732 38631440

[64] Sorokin DY, Merkel AY, Ziganshin RH et al. Growth physiology, genomics, and proteomics of *Desulfurivibrio dismutans* sp. nov., an obligately chemolithoautotrophic, sulfur disproportionating and ammonifying haloalkaliphile from soda lakes. *Front Microbiol* 2025;16:1590477. 10.3389/fmicb.2025.1590477 40485835 PMC12142623

[65] Novak LVF, Jiang L, Hemon M et al. Sulfur disproportionation occurs globally across anoxic habitats and has multiple mechanisms of independent evolutionary origin. *ISME J* 2026;20:wrag042. 10.1093/ismejo/wrag042 41766620 PMC12998232

[66] Rost B . Twilight zone of protein sequence alignments. *Prot Eng* 1999;12:85–94. 10.1093/protein/12.2.85 10195279

