# Supplementary material for: Distribution of a novel DsrEFH sulfur transferase suggests widespread sulfur oxidation capacity in sulfate reducers
Source: ISME J. 2026 May 23;20(1):wrag130. doi: 10.1093/ismejo/wrag130 (PMC13293266; doi:10.1093/ismejo/wrag130)
Supplement: Supplementary_material_wrag130 [file supplementary_material_wrag130.zip › Plum-Jensen_ISME2026-SI-final-accepted_wrag130.pdf]

Distribution of a novel DsrEFH sulfur transferase suggests widespread sulfur oxidation capacity in sulfate reducers

Lea E. Plum-Jensen, Marc G. Mohr, Tomohisa Sebastian Tanabe, Bo Wang, Simon G. Echers, Nikoline S. Madsen, Casper Thorup, Markéta Linhartová, Lars Peter Nielsen, Morten K.D. Dueholm, Thomas Boesen, Ian P.G. Marshall, Christiane Dahl, and Andreas Schramm

Supplementary Information

Supplementary Methods

Supplementary Discussion

References

Supplementary Figures:

Fig. S1: Expression of the TusA/DsrEFH operon in Electronema aureum GS

Fig. S2: AlphaFold2 structure predictions of the putative DsrEFH type II monomers

Fig. S3: AlphaFold2 structural prediction of the putative DsrEFH type II heterotrimer, and alignment to known DsrEFH type I from A. vinosum

Fig. S4: Sulfur transfer between TusA and DsrEFH or DsrE(Cys^{67}Ser)FH

Fig. S5: Sulfur transfer between DsrEFH or DsrE(Cys^{67}Ser)FH and DsrC

Fig. S6: Sulfur transfer between TusA and DsrC

Fig. S7: Persulfidation test with thiosulfate ($S_2O_3^{2-}$)

Fig. S8: The phylogenetic relationship of DsrEFH type II sequences

Fig. S9: Proposed pathways for disproportionation of sulfite and elemental sulfur

Supplementary Tables:

Table S1: Genomes included in the pangenome-analysis

Table S2: Strains, plasmids and primers

Table S3: Comparison of the structures and sequences of DsrEFH type II from *Electronema aureum* GS to a known structure of DsrEFH type I from *Allochromatium vinosum* (RCSB ID=2HY5)

Table S4: Detection of protein masses after sulfur transfer reactions between TusA, DsrC and DsrEFH-N-Strep

Table S5: Bacterial isolates that contain the dsrEFH type II genes

Table S6: Sulfur disproportionating bacterial species not retrieved by HMM search for DsrEFH type II

Additional data supplements (separate excel files):

Data Supplement 1: Core genes of the cable bacterial pangenome

Data Supplement 2: Most highly expressed genes from cable bacteria in the transcriptome and proteomes analyzed.

Transcriptome data:

Available from NCBI under the accession number PRJNA575166.

Proteome data:

Available from the MassIVE repository (https://massive.ucsd.edu/ProteoSAFe/static/massive.jsp?redirect=auth):

1. *Electrothrix communis* RB enrichment.

   MassIVE dataset: MSV000099163

2. *Electronema aureum* GS enrichment.

   MassIVE dataset: MSV000099164

3. Fractionated protein extraction from *Electronema aureum* GS

   MassIVE dataset: MSV000099166

Supplementary Methods

Proteome Analyses

Whole filament protein extraction of *E. aureum* GS and *E. communis* RB

Single-strain enrichments of *E. aureum* GS (Thorup et al. 2021) and *E. communis* RB (Plum-Jensen et al. 2024) were cultivated in autoclaved sediment as described previously. Tufts of cable bacteria filaments were collected from the sediment under microscopic guidance with very fine tweezers, washed briefly in autoclaved seawater, and collected into a 2 mL Eppendorf tube kept on ice. A total of 0.5 mL of filaments per cable bacterium species was sampled, snap-frozen in liquid nitrogen, and stored at -80°C until sample preparation for mass spectrometry using the iST kit (PreOmics) according to the manufacturers protocol, with minor modifications described below.

Fractional protein extraction of *E. aureum* GS

A sequential protein extraction protocol was developed in this study to improve protein recovery from *E. aureum* GS by a combination of chemical and mechanical disruption to release soluble proteins, followed by detergent treatments of the cell pellets to extract membrane-associated proteins. Tufts of cable bacteria filaments (7x approx. 0.5 mL) were collected as described above, washed at least ten times in autoclaved freshwater to remove attached particles, transferred into seven 2 mL Lysing Matrix E tubes (MP Biomedicals™) with 500 µL of 50 mM Tris-HCl (pH 8) and 150 µL EDTA-free protease inhibitor (cOmplete™, Merck, Germany), snap-frozen in liquid nitrogen and stored at -80°C until further lysis.

For filament lysis, 200 µL B-PER reagent (Thermo Fischer) was added to each sample and incubated for 15 min at room temperature. Samples were then bead-beaten (4 × 20 s at 6.0 m s-1 on a FastPrep-24 instrument), cooled on ice for 2 min, and centrifuged with 14.1k rcf for 15 min at 4°C. The resulting supernatants were pooled and snap-frozen in liquid nitrogen. The pellets were re-extracted with 200 µL SDS loading buffer (2% sodium dodecyl sulfate (SDS), 1% β-mercaptoethanol, 0.04‰ bromophenol blue, 6% glycerol in 50 mM Tris-HCl pH 6.8) by vortexing for 30 s, incubation at 95°C for 5 min, vortexing for 30 s, and centrifugation (14.1k rcf, 15 min, 4°C). Supernatants were again pooled and snap-frozen in liquid nitrogen, while pellets were re-extracted with 8 M urea in SDS loading buffer at room temperature for 10 min, followed by centrifugation. Supernatants were again pooled and snap-frozen in liquid nitrogen. Pellets were finally re-extracted with 200 µL formic acid (100%) at 50°C for 10 min in the fume hood. Small aliquots from each supernatant and the final pellet were used for protein quantification and SDS-PAGE, using bovine serum albumin (BSA) as the standard. Supernatants and pellets were snap-frozen in liquid nitrogen, lyophilized overnight, and stored at -80°C for mass spectrometry. Lyophilized samples were dissolved in 150 µL Milli-Q water and precipitated with 850 µL ice-cold acetone at -20°C overnight. After centrifugation (14.1k rcf, 20 min, 4°C), the pellet was washed with 500 µL ice-cold acetone, sonicated (sonicator bath, 15 min), incubated at -20°C for 2 hours, centrifuged (14.1k rcf, 20 min, 4°C), before the supernatant was discarded and the pellet was air-dried.

Sample preparation for bottom-up proteomics

Acetone-precipitated supernatants were resolubilized in 50 µL "Lyse Buffer" (iST Kit, PreOmics, Germany) and heated to 90°C for 10 min. Subsequently, the solution was transferred to 50 µL AFA tubes (Covaris, Wobum, MA, USA) and subjected to two cycles of the "Protein Extraction" protocol using an M220 focused ultrasonicator (Covaris, Wobum, MA, USA) as previously described (Pedersen et al. 2025). 10 µL sample aliquots before and after ultrasonication were stored for validation by SDS-PAGE analysis. The remaining solution (30 µL) was reduced,

alkylated, digested (1.5 h), and cleaned as described by the iST kit manufacturer. After cleanup, digests were dried by SpeedVac (Thermo) and resuspended in 20 µL "Load Buffer". Concentration estimation was performed by Nanodrop (A280, 1A = 1 mg mL^{-1}) and samples diluted as required. Approximately 1 µg cleaned digest was used per analysis by LC-MS/MS.

For the pellet, a similar approach was applied with minor modifications. 50 mg was suspended in 100 µL "Lyse Buffer", heated, transferred to AFA tubes and subjected to four extraction cycles. An increased number of extraction cycles was found beneficial due to the more recalcitrant nature of the matrix, according to SDS-PAGE analysis (data not shown). The remaining steps were identical to those described for acetone-precipitated supernatants.

For the whole filament cable bacteria samples of *E. aureum* GS and *E. communis* RB, 25 µL liquid sample was mixed with 25 µL "2x Lyse Buffer" (Preomics) and subjected to the same extraction, reduction, alkylation, and clean-up procedure as the pellet sample above.

Mass spectrometry and data analysis

All samples were run on a Q Exactive™ HF hybrid quadrupole-Orbitrap mass spectrometer (ThermoFisher Scientific) with an ultra-high-field Thermo Scientific™ Orbitrap™ detector as described elsewhere (Echers et al. 2022). Mass spectrometry data were analyzed with MaxQuant v.2.2.0.0 (Cox & Mann 2008; Tyanova et al. 2016) with parameters fitting the sample preparation, label-free quantification, and a false discovery rate = 1%, and the data were searched against proteomes translated from the high quality genomes of *E. aureum* GS (Sereika et al. 2023) and *E. communis* RB (Plum-Jensen et al. 2024).

Recombinant protein production and sulfur transfer experiments

Plasmid design

The synthesized DNA fragments contained an NcoI recognition site at the 5' end and a BamHI recognition site at the 3' end. In addition, a strep-tag-encoding sequence was fused either to the 5' or the 3' end. The fragments were placed between the NcoI and BamHI sites of pET28a, resulting in pET28a-tusA-C-Strep, pET28a-dsrC-N-Strep, pET28a-dsrEFH-N-Strep and pET28a-dsrEFH-C-Strep.

Recombinant TusA, DsrC, and DsrEFH type II protein production

Overnight precultures of *E. coli* BL21(DE3) were inoculated in fresh LB medium at 5% (v/v). Synthesis of the recombinant proteins was induced by the addition of 1 mM IPTG when cultures reached an OD600 of 0.6-0.8, followed by incubation at 37°C for 2.5 h. Cells were harvested by centrifugation (11,000 × g, 20 min, 4°C) and resuspended in 50 mM Tris-HCl buffer (pH 7.5) containing 150 mM NaCl. Cells were lysed by sonication. Insoluble cell material was then removed by centrifugation (16,100 × g, 30 min, 4°C). Strep-tagged proteins were purified using Strep-Tactin Superflow (IBA Lifesciences, Göttingen, Germany) according to the manufacturer's instructions. Proteins were then transferred to salt-free 50 mM Tris-HCl buffer (pH 7.5) and stored at -70°C. Size exclusion chromatography on HiLoad 16/60 Superdex™ 75 (Cytiva, Freiburg, Germany) was performed as previously described (Li et al. 2023).

Supplementary Discussion

DsrEFH type II as potential marker for S disproportionation

When the *yeeE/yedE-tusA-dsrEFH* type II (formerly YTD) gene cluster was first described, it was hypothesized that it was essential for sulfur disproportionation, but without proposing a specific

function in the disproportionation pathway (Umezawa et al. 2020). Later, the upregulation of these genes in *Desulfolithobacter dissulfuricans* during growth by thiosulfate disproportionation lend further support for this proposal (Hashimoto et al., 2022). However, it is difficult to see how the YTD cluster or DsrEFH type II could be a universal marker for disproportionation: if DsrEFH type II was generally required for the disproportionation of sulfur compounds, it would be expected to occur in all organisms capable of this metabolism, and all DsrEFH type II-containing microbes would be able to disproportionate. This is clearly not the case (Table S5 & S6, see also Novak et al. 2026), even if we consider that some of the genomes in these lists are incomplete and genes may thus have been missed, or that not all DsrEFH type II-containing microbes have been experimentally tested for disproportionation: some disproportionators do not involve the DSR pathway at all, for example *Desulfurella amilsii* for S^0 disproportionation (Florentino et al. 2016) and sulfur disproportionators belonging to the phylum Campylobacterota (Wang et al. 2022).

Although not a universal marker, DsrEFH type II could theoretically still be a marker for specific disproportionation pathways: different sulfur species with intermediate redox states can be disproportionated, including sulfite, elemental sulfur and thiosulfate (Figure S9a-c). In the disproportionation of sulfite, the DSR system supposedly fulfills a reductive role and thus involvement of DsrEFH type II is unlikely (Supplementary Fig. S9a); thiosulfate disproportionation likely occurs independently of the Dsr system, potentially involving rhodaneses (Supplementary Fig. S9c), which have been identified as key enzymes in sulfur disproportionation in *Desulfurella amilsii* (Florentino et al. 2018). Consistent with these considerations, the presence of *dsrEFH* type II was neither associated with sulfite nor thiosulfate disproportionation (Supplementary Table S6).

In contrast, disproportionation of elemental sulfur requires oxidation of sulfane sulfur, and thus for its oxidative branch might involve the sulfur transferases TusA and DsrEFH type II (Supplementary Fig. S9b; Sorokin et al. 2025). However, the occurrence of *dsrEFH* type II among sulfur disproportionators did not correlate with the ability to disproportionate S^0 either (Supplementary Table S6).

In this light, the upregulation of the YTD gene cluster (incl. DsrEFH type II) in *Desulfolithobacter dissulfuricans* during growth by thiosulfate disproportionation (Hashimoto et al., 2022) is hard to explain; however, it is unknown how gene expression for sulfur disproportionation is regulated: could the presence of any intermediate sulfur compound (here: thiosulfate) induce all necessary pathways (here: including the YTD-containing pathway for S^0 disproportionation)?

In conclusion, although it is still possible that *dsrEFH* type II is involved in the oxidation reaction of certain sulfur disproportionators, it cannot be a universal marker for sulfur disproportionation nor a specific marker for a specific sulfur disproportionation pathway.

References

Cox, J. and M. Mann. 2008. MaxQuant enables high peptide identification rates, individualized p.p.b.-range mass accuracies and proteome-wide protein quantification. Nature Biotechnol. 26 (12): 1367-1372. doi: 10.1038/nbt.1511.

Echers, S.G., N. Abdul-Khalek, R.K. Mikkelsen, S.L. Holdt, C. Jacobsen, E.B. Hansen, T.H. Olsen, J.J. Sejberg, and M.T. Overgaard. 2022. Is Gigartina a potential source of food protein and functional peptide-based ingredients? Evaluating an industrial, pilot-scale extract by

proteomics and bioinformatics. Future Foods 6: 100189. https://doi.org/10.1016/j.fufo.2022.100189

Florentino AP, Brienza C, Stams AJM, Sanchez-Andrea I. 2016. *Desulfurella amilsii* sp. nov., a novel acidotolerant sulfur-respiring bacterium isolated from acidic river sediments. *Int. J. Syst. Evol. Microbiol.* 2016; 66(3):1249-1253. https://doi.org/10.1099/ijsem.0.000866

Hashimoto Y, Shimamura S, Tame A, Sawayama S, Miyazaki J, Takai K, and Nakagawa S. 2022. Physiological and comparative proteomic characterization of *Desulfolithobacter dissulfuricans* gen. nov., sp. nov., a novel mesophilic, sulfur-disproportionating chemolithoautotroph from a deep-sea hydrothermal vent. Front. Microbiol. 13:1042116. doi: 10.3389/fmicb.2022.1042116

Li, J., K.Törkel, J. Koch, T.S.Tanabe, H.Y. Hsu, and C. Dahl. 2023. In the Alphaproteobacterium Hyphomicrobium denitrificans SoxR Serves a Sulfane Sulfur-Responsive Repressor of Sulfur Oxidation. Antioxidants 12: 1620. https://doi.org/10.3390/antiox12081620

Novak LVF, Jiang L, Hemon M et al. Sulfur disproportionation occurs globally across anoxic habitats and has multiple mechanisms of independent evolutionary origin. *ISME J* 2026; 20(1): wrag042, 10.1093/ismejo/wrag042

Pedersen, K.D.A., L.T. Andersen, M. Heiselberg, C.A. Brigsted, F.L. Støvring, L.M. Mikkelsen, S.A. Hansen, C.E. Rusbjerg-Weberskov, M. Lübeck, and S. Gregersen Echers. 2025. Identifying Endogenous Proteins of Perennial Ryegrass (Lolium perenne) with Ex Vivo Antioxidant Activity. Proteomes 13: 8. https://doi.org/10.3390/proteomes13010008

Plum-Jensen, L.E., A. Schramm, and I. P. G. Marshall. 2024. First single-strain enrichments of Electrothrix cable bacteria, description of E. aestuarii sp. nov. and E. rattekaaiensis sp. nov., and proposal of a cable bacteria taxonomy following the rules of the SeqCode. Syst. Appl. Microbiol. 47 (1): 126487 https://doi.org/10.1016/j.syapm.2024.126487.

Sereika, M., F. Petriglieri, T.B.N. Jensen, A. Sannikov, M. Hoppe, P.H. Nielsen, I.P.G. Marshall, A. Schramm, M. Albertsen. 2023. Closed genomes uncover a saltwater species of Candidatus Electronema and shed new light on the boundary between marine and freshwater cable bacteria. ISME J. 17: 561–569. https://doi.org/10.1038/s41396-023-01372-6.

Sorokin DY, Merkel AY, Ziganshin RH and Kublanov IV. 2025. Growth physiology, genomics, and proteomics of *Desulfurivibrio dismutans* sp. nov., an obligately chemolithoautotrophic, sulfur disproportionating and ammonifying haloalkaliphile from soda lakes. *Front. Microbiol.* 16:1590477. doi: 10.3389/fmicb.2025.1590477

Thorup, C. Petro, A. Bøggild, T.S. Ebsen, S. Brokjær, L.P. Nielsen, A. Schramm, and J.J. Bjerg. 2021. How to grow your cable bacteria: Establishment of a stable single-strain culture in sediment and proposal of Candidatus Electronema aureum GS. Syst. Appl. Microbiol. 44 (5): 126236-126236. 10.1016/j.syapm.2021.126236.

Tyanova, S., T. Temu, and J. Cox. 2016. The MaxQuant computational platform for mass spectrometry-based shotgun proteomics. Nature Protocols 11 (12): 2301-2319. 10.1038/nprot.2016.136.

Umezawa, K., H. Kojima, Y. Kato, and M. Fukui. 2020. Disproportionation of inorganic sulfur compounds by a novel autotrophic bacterium belonging to Nitrospirota. Systematic and Applied Microbiology 43(5): 126110. doi: https://doi.org/10.1016/j.syapm.2020.126110.

Wang, S., Jiang, L., Xie, S., Alain, K., Wang, Z., Wang, J., Liu, D. & Shao, Z. 2023. Disproportionation of inorganic sulfur compounds by mesophilic chemolithoautotrophic Campylobacterota. Msystems, 8(1), e00954-22.

Supplementary Figures

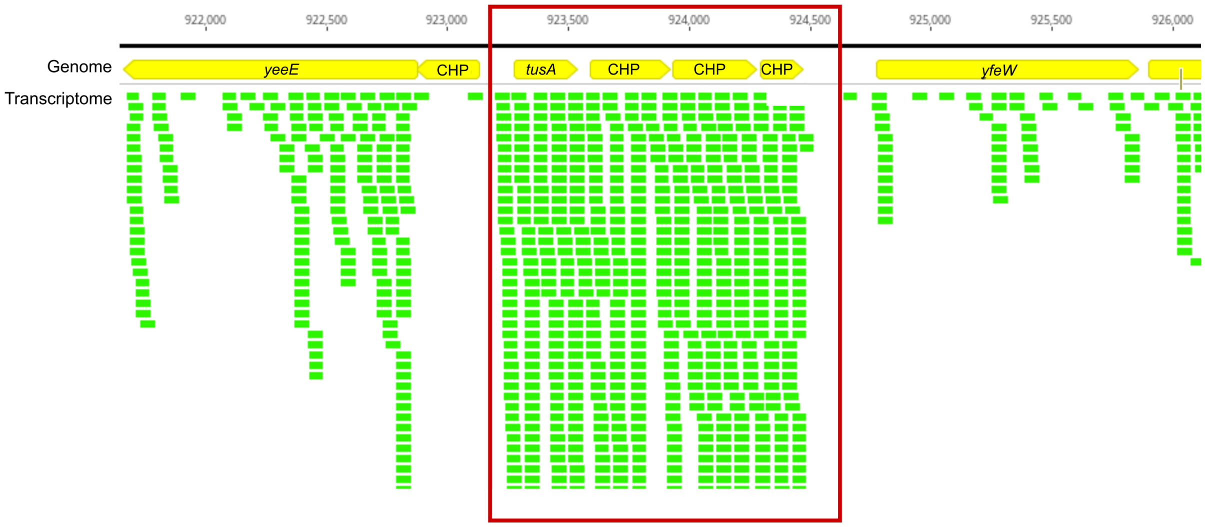

Figure S1: Expression of the TusA/DsrEFH operon in *Electronema aureum* GS. Transcriptomic reads are mapped to the region and shown in green bars. CHP = conserved hypothetical protein.

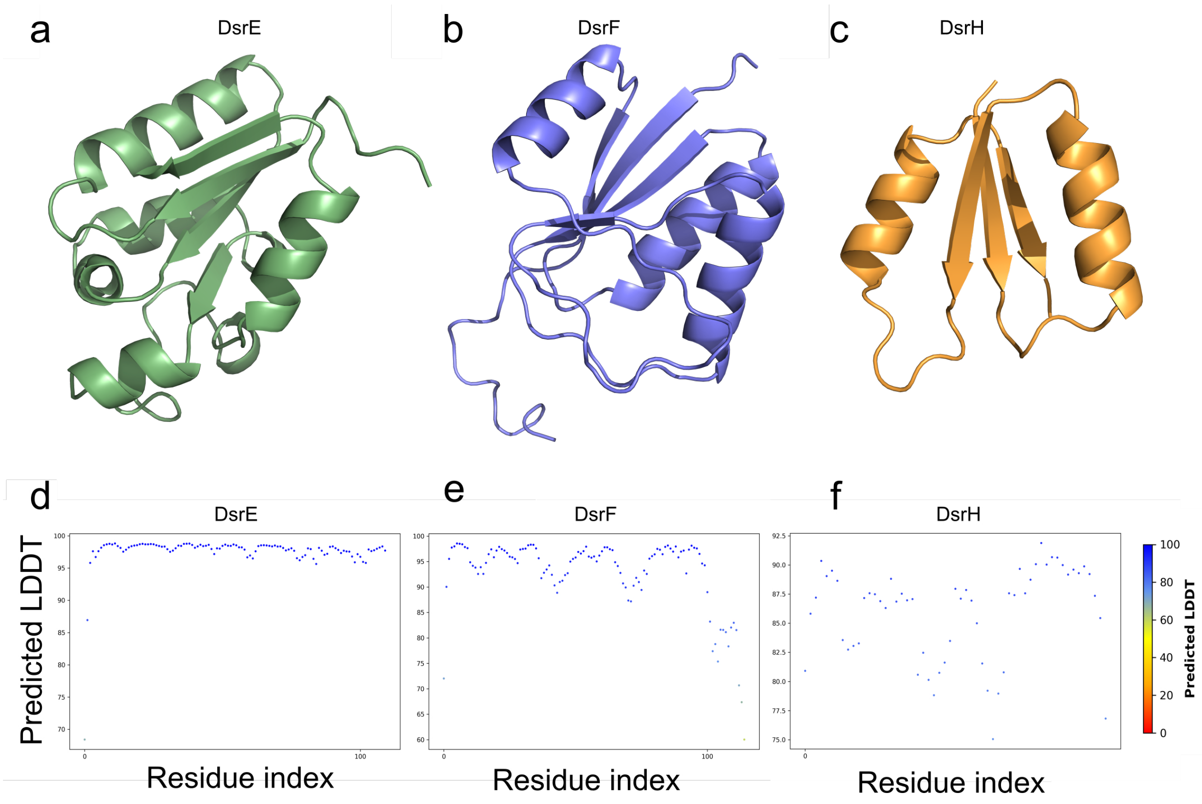

Figure S2: AlphaFold2 structure predictions of the putative DsrEFH type II monomers. a, DsrE type II, **b,** DsrF type II, **c,** DsrH type II. **d-f,** predicted local distance difference test (pLDDT) of the monomers DsrE, DsrF, and DsrH type II.

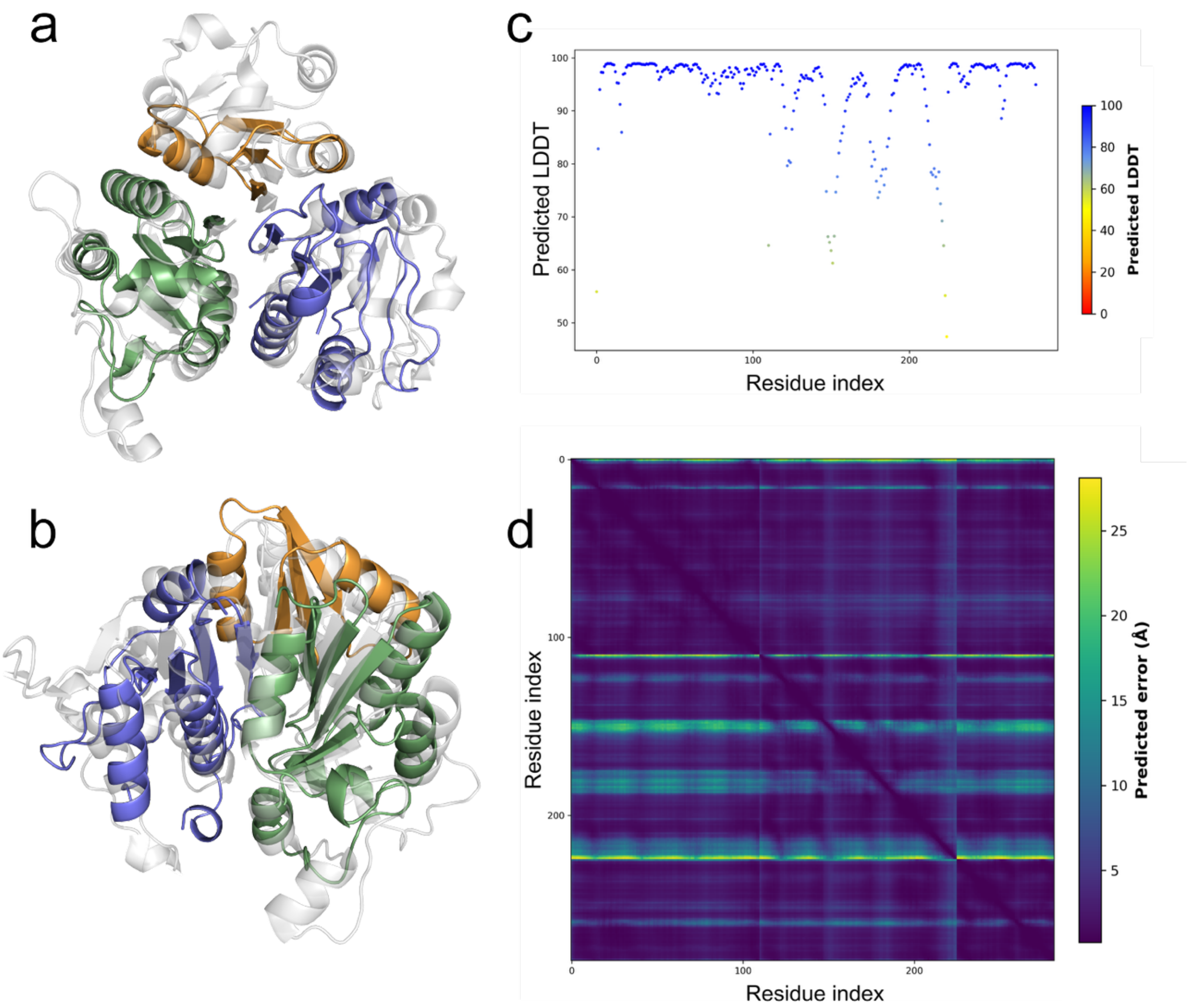

Figure S3: AlphaFold2 structural prediction of the putative DsrEFH type II heterotrimer, and alignment to known DsrEFH type I from *Allochromatium vinosum*. a, top view of alignment, **b,** side-view of alignment. **c,** predicted local distance difference test (pLDDT) and **d,** predicted error (PAE) of the AlphaFold2 prediction.

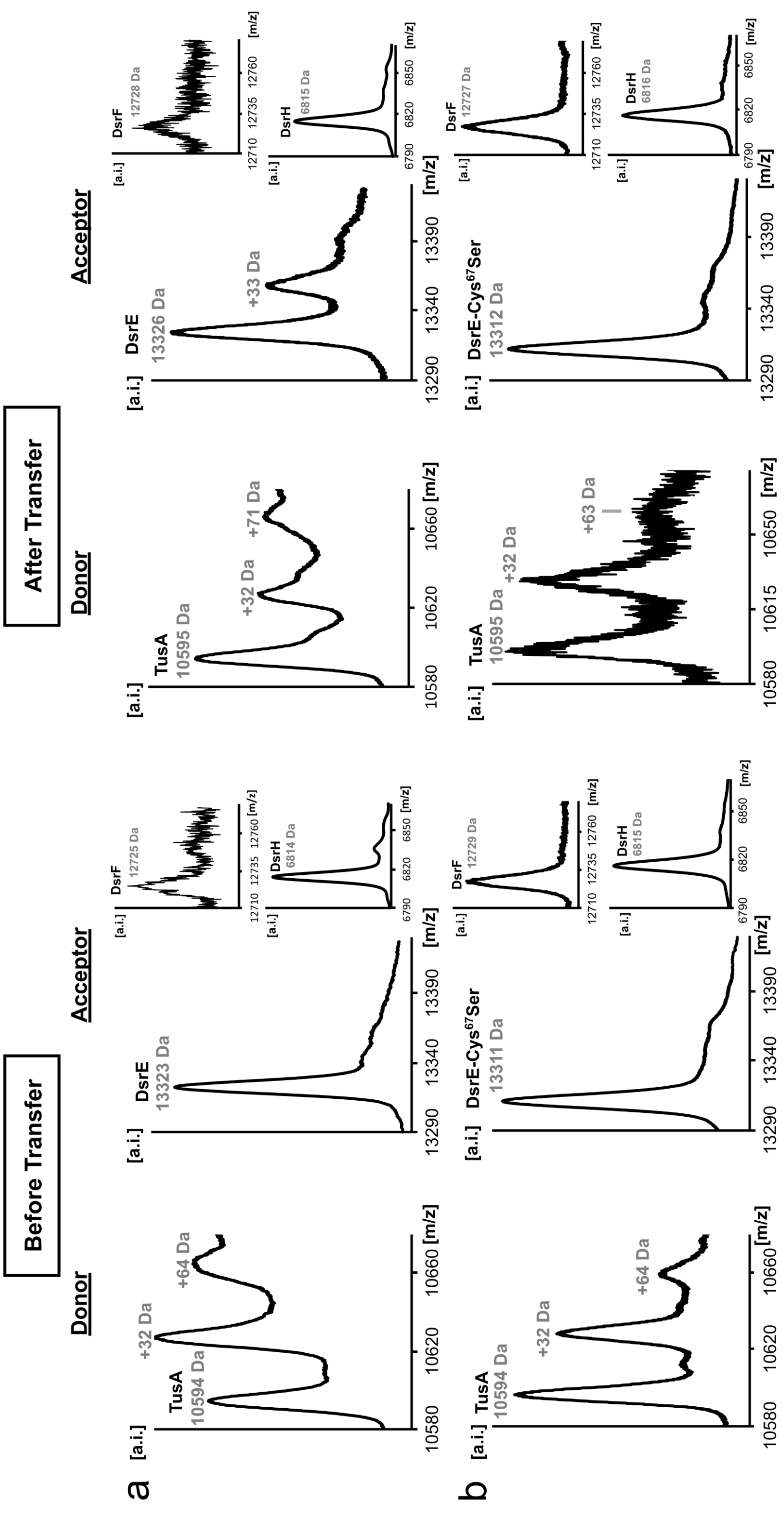

Figure S4 (page 9-10): Sulfur transfer between TusA and DsrEFH or DsrE(Cys^{67}Ser)FH. Mass spectra are provided for each protein before (left panels) and after (right panels) the reaction of persulfidated donor and acceptor. Results are shown for transfer from **a,** TusA to DsrEFH, **b,** TusA to DsrE(Cys^{67}Ser)FH.

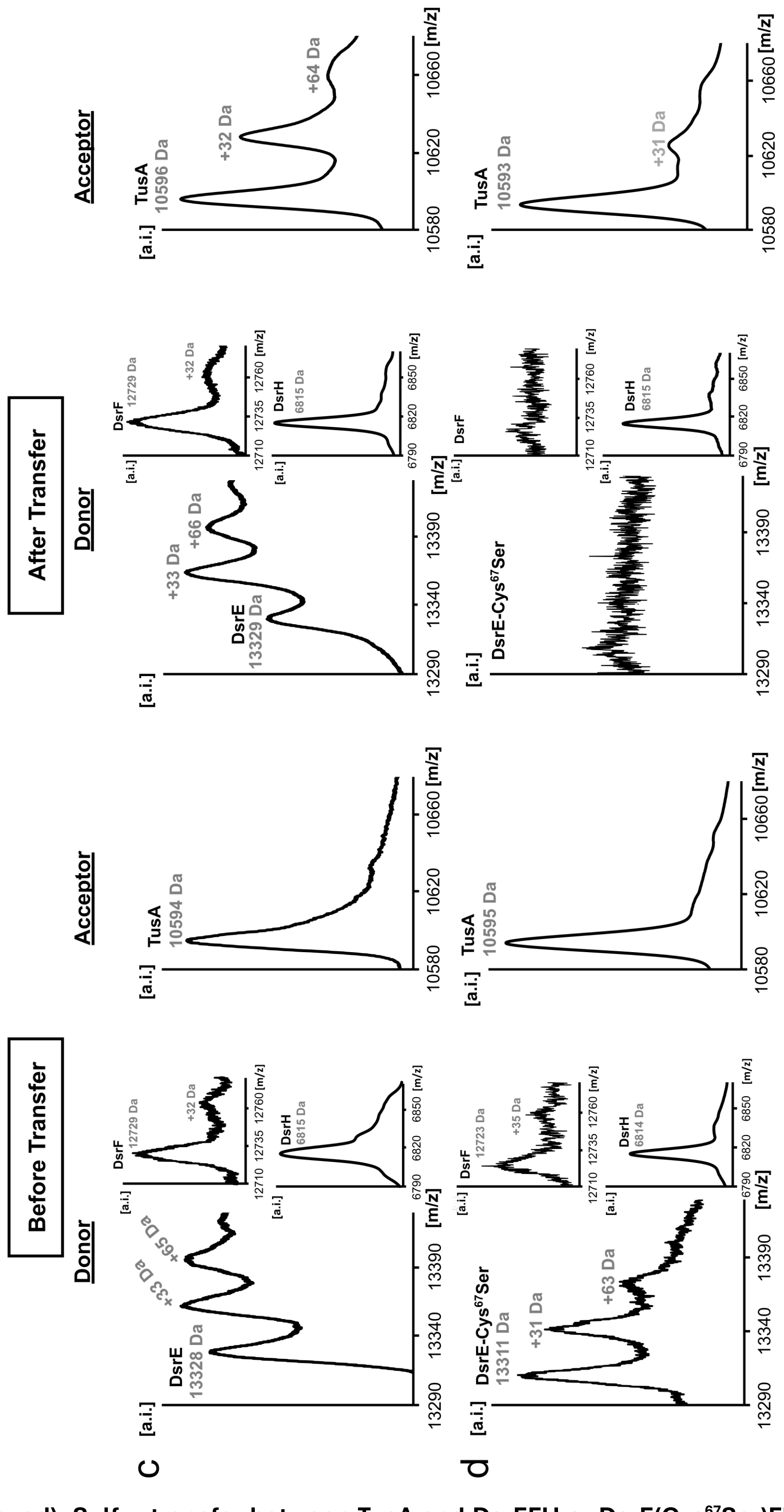

Figure S4 (continued): Sulfur transfer between TusA and DsrEFH or DsrE(Cys^{67}Ser)FH. Mass spectra are provided for each protein before (left panels) and after (right panels) the reaction of persulfidated donor and acceptor. Results are shown for transfer from **c,** DsrEFH to TusA, and **d,** DsrE(Cys^{67}Ser)FH to TusA. A mass increase of 32 Da corresponds to a single persulfidation. Note that persulfidated DsrE-Cys^{67}Ser and DsrF evaded detection after incubation with TusA (panel d). However, this does not prevent the conclusion that there is hardly any sulfur transfer from the DsrE(Cys^{67}Ser)FH variant to TusA. Although MALDI-TOF mass spectrometry does not allow direct quantification, it is obvious that the ratio of native to persulfidated TusA peaks is orders of magnitude higher when the variant DsrEFH protein acts as a sulfur donor (compare the rightmost graphs in panels c and d).

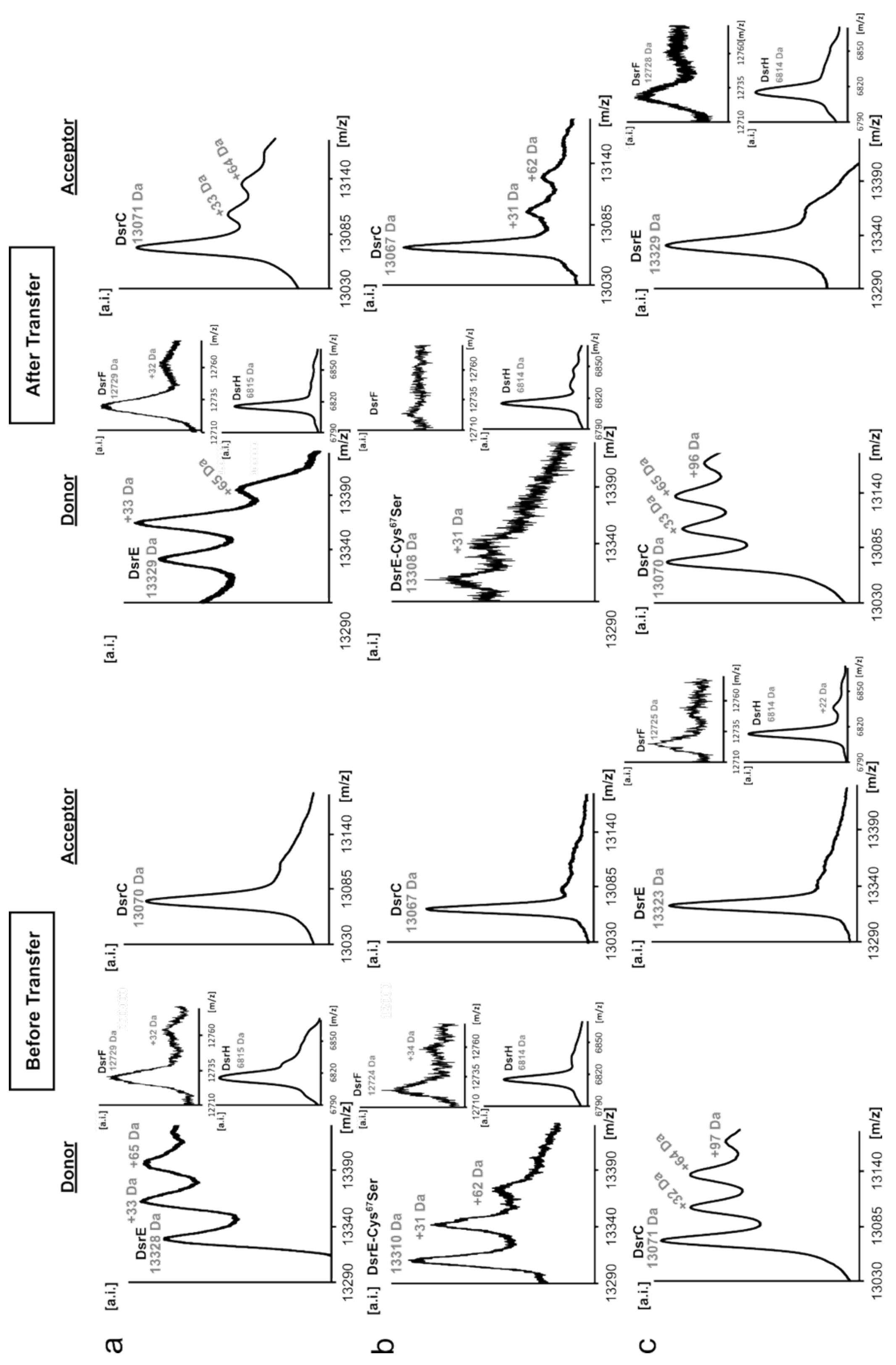

Figure S5: Sulfur transfer between DsrEFH or DsrE(Cys^{67}Ser)FH and DsrC. Mass spectra are provided for each protein before (left panels) and after (right panels) the reaction of persulfidated donor and acceptor. Results are shown for transfer from **a,** DsrEFH to DsrC, **b,** DsrE(Cys^{67}Ser)FH to DsrC, and **c,** DsrC to DsrEFH. A mass increase of 32 Da corresponds to a single persulfidation.

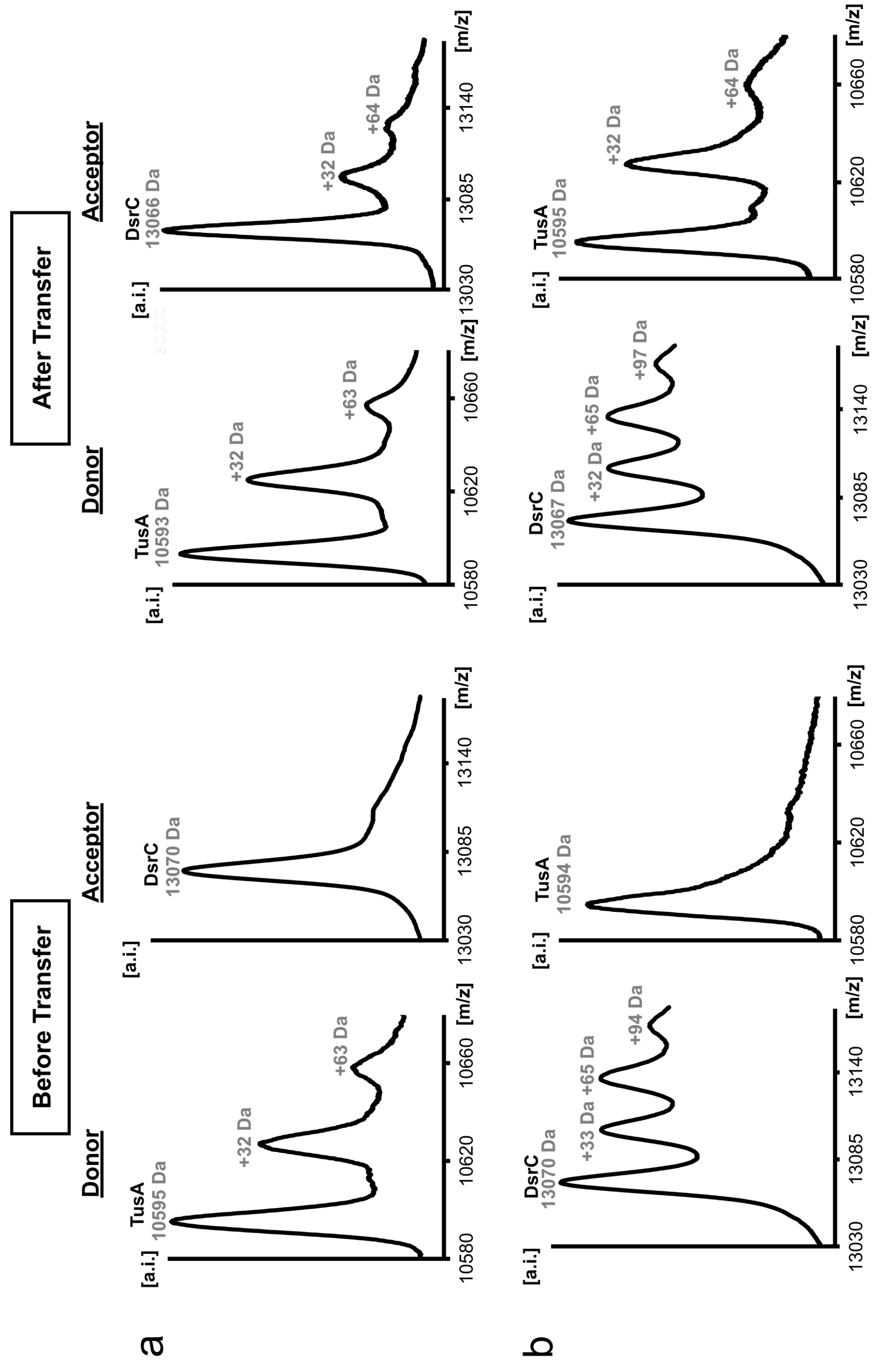

Figure S6: Sulfur transfer between TusA and DsrC. Mass spectra are provided for each protein before (left panels) and after (right panels) the reaction of persulfidated donor and acceptor. Results are shown for transfer from **a,** TusA to DsrC and **b,** DsrC to TusA. A mass increase of 32 Da corresponds to a single persulfidation.

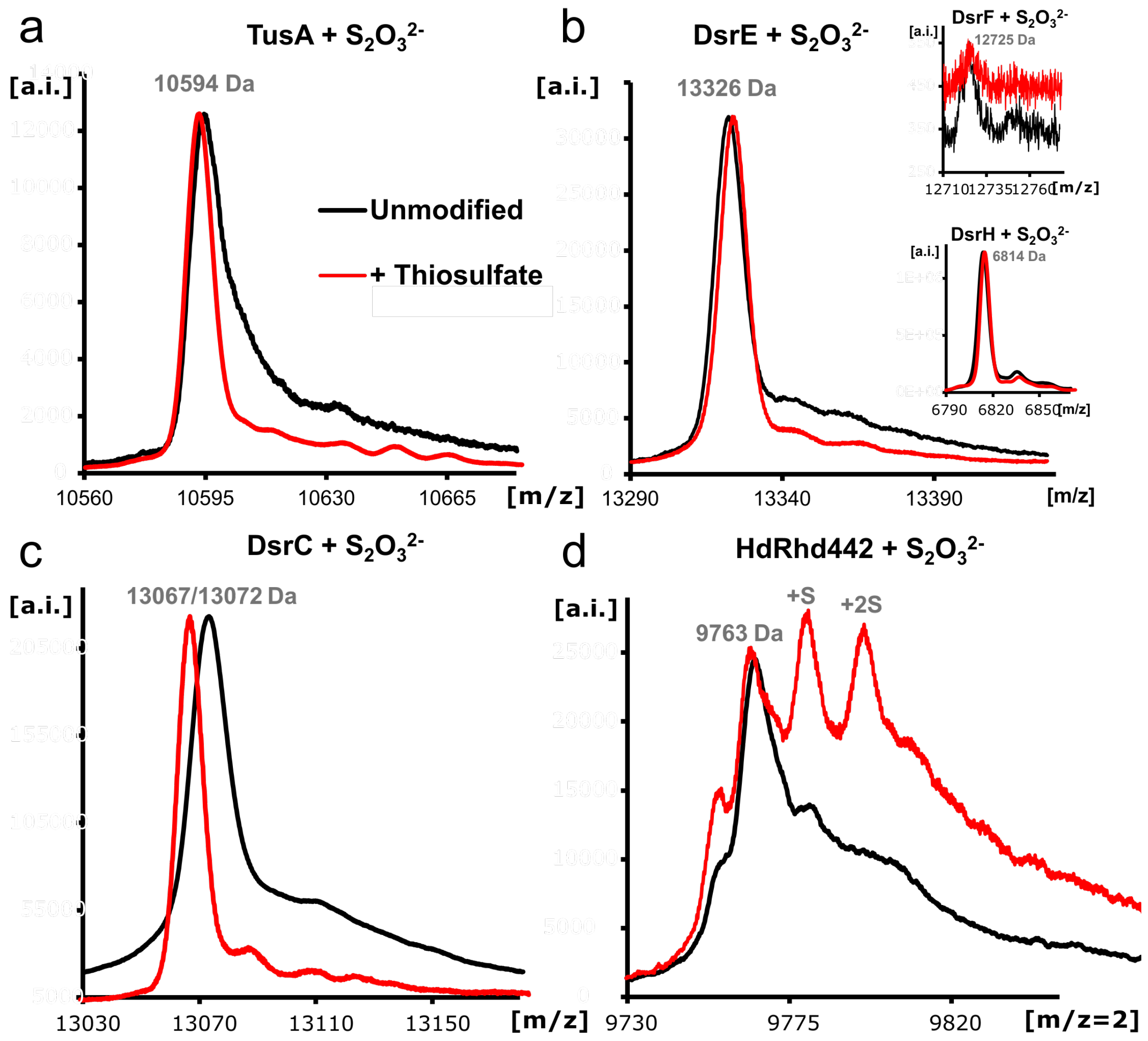

Figure S7: Persulfidation test with thiosulfate ($S_2O_3^{2-}$). Mass spectra are provided for each protein before (black line) and after (red line) incubation with 1 mM thiosulfate. Results are shown for **a,** cable bacterial TusA, **b,** cable bacterial DsrEFH type II, **c,** cable bacterial DsrC and **d,** the rhodanese Rhd442 from *Hyphomicrobium denitrificans* used as a positive control (Reference: https://doi.org/10.1002/pro.5014). A mass increase of 32 Da corresponds to a single persulfidation (+S).

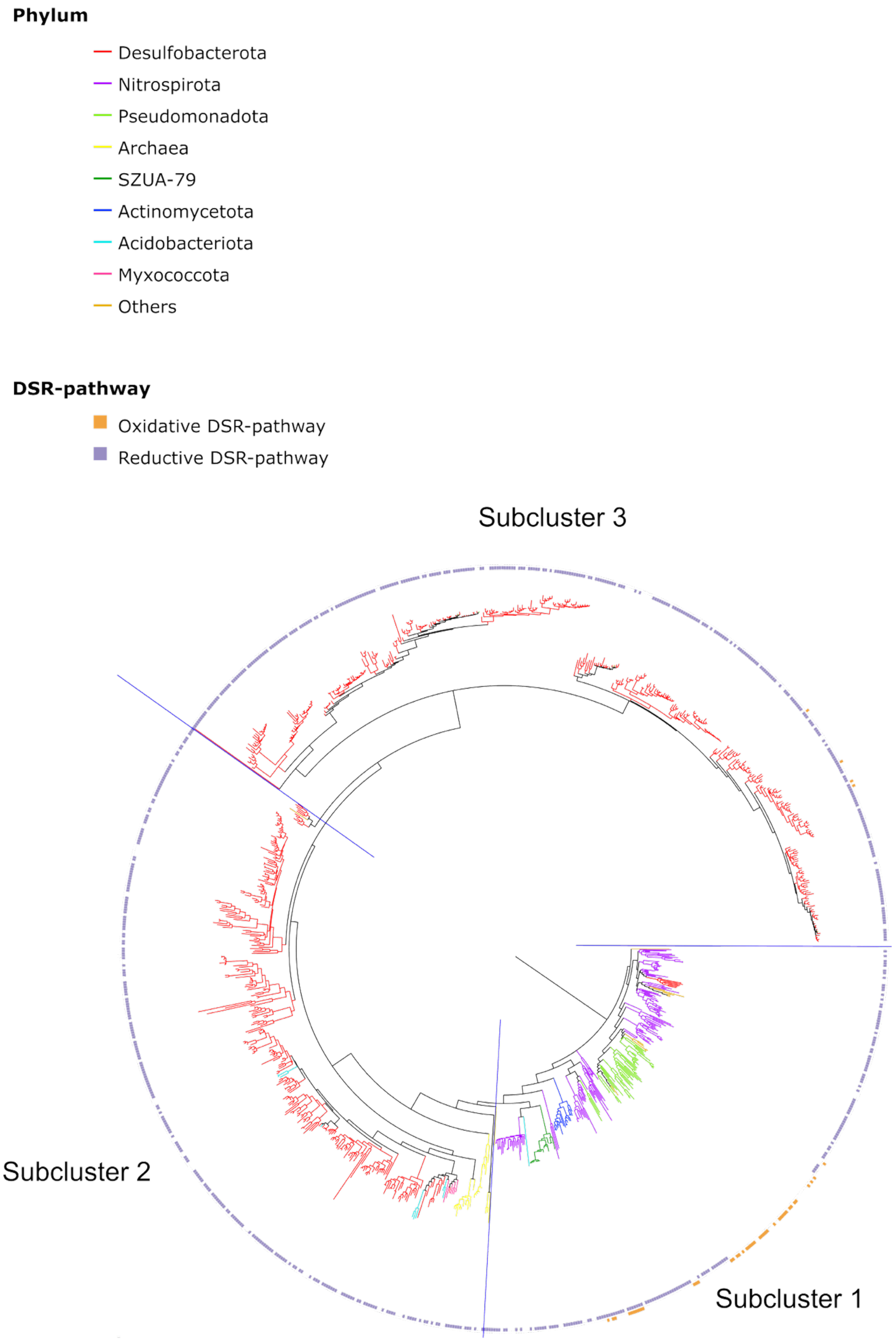

Figure S8: Phylogeny of DsrEFH type II. The 985 DsrEFH type II amino acid sequences were concatenated and aligned, and a ML tree was calculated using IQtree (model LG+R9). For each sequence, the phylum is indicated by a colored node, and the co-occurrence of reductive (grey square) or oxidative (orange square) DsrAB is indicated in the outer circle. Blue lines indicate the three subclusters identified in Fig.5 (main text).

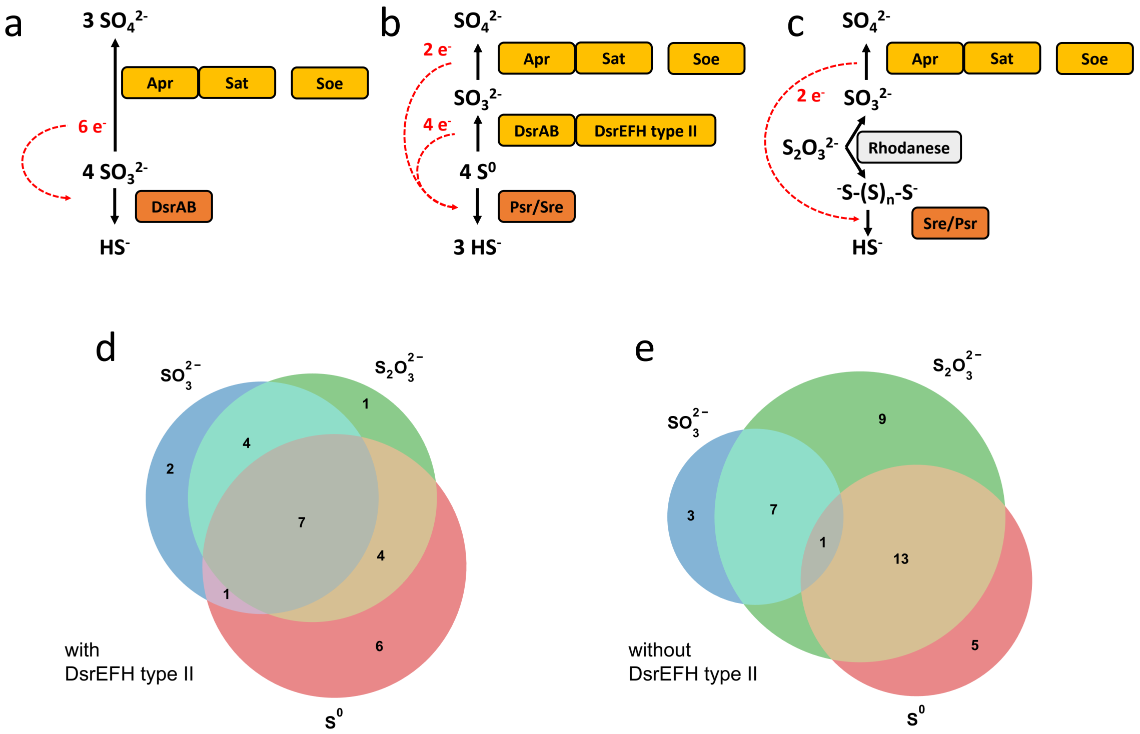

Figure S9: Proposed pathways for disproportionation of sulfite (a), elemental sulfur (b), and thiosulfate (c). Enzymes operating in the oxidative direction are highlighted by yellow boxes, while enzymes catalyzing reductive steps are illustrated in orange. Psr, polysulfide reductase, Sre, sulfur reductase. **(d)** Venn diagram illustrating the distribution of disproportionated sulfur species among sulfur-disproportionating organisms that harbor *dsrEFH* type II and **(e)** lack dsrEFH type II. A comprehensive list of all known sulfur-disproportionating organisms and their ability to disproportionate sulfite (SO_3^{2-}), thiosulfate ($S_2O_3^{2-}$), or elemental sulfur (S^0) is provided in Table S6.

Supplementary Tables

**Table S1: Genomes included in the pangenome-analysis**				
**Genome accession**	**Species name**	**Completeness (%)***	**#contigs**	**Genome size (Mbp)**
GCA_942492785.1	*Electronema aureum* ENR-cMAG	95.27	1 (circular)	2.9
GCA_942493095.1	*Electronema halotolerans* BRK-cMAG	94.35	1 (circular)	3.2
GCA_030644725.1	*Electrothrix communis* RB	95.93	1 (circular)	4.8
GCA_037901825.1	*Electrothrix gigas* HY10-6	94.29	1 (circular)	3,6
GCA_942492895.1	*Electrothrix laxa* MAR-scMAG	90.05	1 (linear)	4.1
GCA_037902255.1	*Ca.* Electrothrix sp. GW3-4	97.20	1 (circular)	4.6
GCA_033584155.1	*Ca.* Electrothrix scaldis GW3-3**	96.70	1 (circular)	5.1

*Completeness calculated by CheckM2

**Same species as *Electrothrix aestuarii* Rat1 (GCA_032595685.2)

**Table S2: Comparison of the structures and sequences of DsrEFH type II from *Electronema aureum* GS to a known structure of DsrEFH type I from *Allochromatium vinosum* (RCSB ID=2HY5)**				
**Protein**	**DALI Z-score**	**RMSD (Å)**	**TM-score**	**Amino acid sequence identity (%)**
DsrE	12.4	2.12	0.71	24.27
DsrF	7.9	2.83	0.60	13.40
DsrH	4.5	2.04	0.69	20.83
DsrEFH	12.3	3.27	0.75	21.90

**Table S3: Strains, plasmids and primers**		
**Strain, plasmid or primer**	**Relevant genotype, description or sequence**	**Reference or source**
**Strains**		
E. coli DH5α	F^- ϕ80*lac*ZΔM15 Δ(*lac*ZYA-*arg*F)U169 *rec*A1 *end*A1 *hsd*R17(r_K^-, m_K^+) *pho*A *sup*E44 λ^-*thi*-1 *gyr*A96 *rel*A1	New England Biolabs
E. coli BL21 (DE3)	F^-*omp*T *hsd*S_B (r_B^-, m_B^-) *gal dcm* (DE3)	Novagen
**Plasmids**		
pET28a-*tusA*-C-Strep	Km^r, pET-28a with *tusA*-C-Strep insertion between NcoI and BamHI	This work
pET28a-*dsrC*-N-Strep	Km^r, pET-28a with *dsrC*-N-Strep insertion between NcoI and BamHI	This work
pET28a-*dsrEFH*-C-Strep	Km^r, pET-28a with *dsrEFH*-C-Strep insertion between NcoI and BamHI	This work
pET28a-*dsrEFH*-N-Strep	Km^r, pET-28a with *dsrEFH*-N-Strep insertion between NcoI and BamHI	This work
pET28a-*dsrEFH*-Cys^{67}Ser-N-Strep	Km^r, pET-28a with *dsrEFH*-Cys^{67}Ser-N-Strep insertion between NcoI and BamHI	This work
**Primers**		
fw_*dsrE*_Cys^{67}Ser	TCGCCATTTCCGCGGATTCC	This work
rev_*dsrE*_Cys^{67}Ser	TATCTTCTTCACCGCAGAGC	This work

Table S4: Detection of protein masses after sulfur transfer reactions between TusA, DsrC and DsrEFH-N-Strep. Donor proteins were persulfidated by incubation with polysulfide. After removal of free polysulfide, persulfidated donors were mixed with the acceptor proteins and incubated for 30 min, followed by mass spectrometric analysis of the proteins mixtures. Calculated masses are given without initiator methionines except for DsrF and DsrH. Increases of 32±2 Da or multiples thereof indicate persulfidation with one or more sulfur atoms.

Donor protein					Acceptor protein			
Protein	Calculated masses unmodified proteins [Da]	Detected masses [Da]	Modifications		Protein	Calculated masses unmodified proteins [Da]	Detected masses [Da]	Modifications
TusA	10594	10594	-	→	DsrE	13326	13326	-
		10626 (Δ32)	-S				13359 (Δ33)	-S
		10658 (Δ64)	$-S_2$		DsrF	12725	12728	-
					DsrH	6814	6815	-
TusA	10594	10594	-	→	DsrE-Cys^{67}Ser	13310	13312	-
		10626 (Δ32)	-S		DsrF	12725	12727	-
		10658 (Δ64)	$-S_2$		DsrH	6814	6816	-
DsrE	13326	13328	-	→	TusA	10594	10596	-
		13361 (Δ33)	-S				10628 (Δ32)	-S
		13393 (Δ65)	$-S_2$				10660 (Δ64)	$-S_2$
		13424 (Δ96)	$-S_3$					
DsrF	12725	12729	-					
		12761 (Δ32)	- S					
DsrH	6814	6815	-					
DsrE-Cys^{67}Ser	13310	13310	-	→	TusA	10594	10594	-
		13341 (Δ31)	-S					
		13373 (Δ63)	$-S_2$					
DsrF	12725	12723	-					
		12758 (Δ35)	-S					
DsrH	6814	6815	-					
DsrE	13326	13328	-	→	DsrC	13067	13071	-
		13361 (Δ33)	-S				13104 (Δ33)	-S
		13393 (Δ65)	$-S_2$				13135 (Δ64)	$-S_2$
		13424 (Δ96)	$-S_3$					
DsrF	12725	12729	-					
		12761 (Δ32)	-S					
DsrH	6814	6815	-					
DsrE-Cys^{67}Ser	13310	13310	-	→	DsrC	13067	13065	-
		13341 (Δ31)	-S				13096 (Δ31)	-S
		13372 (Δ62)	$-S_2$				13127 (Δ62)	$-S_2$
DsrF	12725	12724	-					
		12758 (Δ34)	-S					
DsrH	6814	6814	-					
DsrC	13067	13071	-	→	DsrE	13326	13329	-
		13103 (Δ36)	-S		DsrF	12725	12728	-
		13135 (Δ68)	$-S_2$		DsrH	6814	6814	-
		13168 (Δ101)	$-S_3$				6836 (Δ22)	$-Na^+$
TusA	10594	10594	-	→	DsrC	13067	13066	-
		10626 (Δ32)	-S				13098 (Δ32)	-S
		10657 (Δ63)	$-S_2$				13130 (Δ64)	$-S_2$
DsrC	13067	13070	-	→	TusA	10594	10595	-
		13103 (Δ33)	-S				10627 (Δ32)	-S
		13135 (Δ65)	$-S_2$				10659 (Δ64)	$-S_2$
		13164 (Δ94)	$-S_3$					

Table S5: Bacterial isolates that contain the *dsrEFH* type II genes

Taxonomy, gene content of the *dsrEFH* operon, and the reported physiology of 46 isolated bacterial strains that contain *dsrEFH* type II genes. References and genome accession numbers refer to the publication describing the physiology and related genome. Grey shading indicates strains positive for sulfur disproportionation; a black frame around the physiology information indicates strains positive for sulfur oxidation.

Phylum	Class	Order	Family	Species	Genes	Experimentally confirmed physiology	Reference	Genome accession number
Desulfobacterota								
	Desulfarculia							
		Desulfarculales						
			Desulfarculaceae					
				Desulfarculus baarsii DSM 2075[T]	DsrEFH type II	Sulfate reduced	Widdel and Bak 1992	GCF 000143965
				Desulfocarbo indianensis SCBM[T] (DSM 28127[T])	DsrEFH type II	Sulfate and thiosulfate reduced, sulfite and S^0 not reduced	An and Picardal 2014	GCA 001184205
	Desulfovibrionia							
		Desulfovibrionales						
			Desulfonatronaceae					
				Desulfonatronum thioautotrophicum ASO4-1[T] (DSM 21337[T])	YeeE-TusA-DsrEFH type II	Sulfate, sulfite and thiosulfate reduced, S^0 reduced but without growth, thiosulfate and sulfite dismutated	Sorokin et al. 2011	GCF 000934745
			Desulfohalobiaceae					
				Desulfothermus okinawensis (JCM 13304[T])	TusA-DsrEFH type II type II	Sulfate and thiosulfate reduced	Nunoura et al. 2007	GCA 001311565
			Desulfonatronovibrionaceae					
				Desulfonatronospira thiodismutans ASO3-1[T] (DSM 19093[T])	TusA-TusA-YeeE-DsrEFH type II	Sulfate, sulfite, and thiosulfate reduced, sulfite and thiosulfate disproportionated	Sorokin et al. 2008	GCF 000174435
				Desulfonatronovibrio hydrogenovorans DSM 9292[T]	YeeE-TusA-DsrEFH type II	Sulfate, sulfite, and thiosulfate reduced, S^0 not reduced, thiosulfate dismutated	Zhilina et al. 1997	GCF 000686525

Desulfonatronovibrio magnus AHT22^T (DSM 24400^T)	YeeE-TusA-DsrEFH type II	Sulfate, sulfite and thiosulfate reduced, thiosulfate and sulfite disproportionated	Sorokin et al. 2011	GCF 000934755
Desulfobulbia				
Desulfobulbales				
Desulfobulbaceae				
Desulfobulbus oralis HOT041/ORNL^T (ATCC TSD-125^T)	DsrK-TusA-DsrEFH type II	Sulfate and thiosulfate reduced	Cross et al. 2018	GCF 002952055
Desulfobulbus elongatus DSM 2908^T	YeeE-TusA-DsrEFH type II	Sulfate, thiosulfate and sulfite reduced	Samain et al. 1984	GCF 000621145
Desulfobulbus propionicus DSM 2032^T	YeeE-TusA-DsrEFH type II	Sulfate reduced, sulfide, S^0, sulfite and polysulfide oxidized. S^0 disproportionated	Pagani et al. 2011	GCF 000186885
Desulfobulbus oligotrophicus Prop6^T (DSM 103420^T)	YeeE-TusA-DsrEFH type II	Sulfate, sulfite and thiosulfate reduced, sulfite disproportionated	El Houari et al. 2017	GCF 016446285
Desulfobulbus alkaliphilus DSM 24258^T	YeeE-TusA-DsrEFH type II	Sulfate and sulfite reduced, thiosulfate not reduced, thiosulfate and sulfur reduced without growth	Sorokin et al. 2012	GCF 016918545
Desulfobulbus rhabdoformis DSM 8777^T	YeeE-TusA-DsrEFH type II	Sulfate, sulfite and thiosulfate reduced	Lien et al. 1998	GCF 016919065
Desulfogranum japonicum DSM 18378^T *(previously Desulfobulbus japonicus)*	YeeE-TusA-DsrEFH type II	Sulfate and thiosulfate reduced, sulfite not reduced	Suzuki et al. 2007a	GCF 000429945
Desulfogranum mediterraneum DSM 13871^T *(previously Desulfobulbus mediterraneus)*	YeeE-TusA-DsrEFH type II	Sulfate, sulfite and thiosulfate reduced	Sass et al. 2002	GCF 000429965
Desulfogranum marinum DSM 2058^T *(previously Desulfobulbus marinus)*	YeeE-TusA-DsrEFH type II	Sulfate and thiosulfate reduced	Galushko and Kuever 2020	GCF 016918565
Desulfolithobacter dissulfuricans GF1^T (DSM 111414^T)	YeeE-TusA-DsrEFH type II	Sulfate, sulfite and thiosulfate reduced, S^0, thiosulfate and tetrathionate disproportionated	Hashimoto et al. 2022	GCF 025998535

Desulfocapsaceae				
*Desulfocapsa sulfexigens (*DSM 10523^T)	YeeE-TusA-DsrEFH type II	Sulfate not reduced, S^0 and thiosulfate disproportionated	Finster et al. 1998	GCF 000341395
Desulfofustis limnaeus PPLL^T (DSM 110475^T)	YeeE-TusA-DsrEFH type II	Sulfate, thiosulfate, sulfite and S^0 reduced	Watanabe et al. 2022	GCF 023169885
Desulfofustis glycolicus DSM 9705^T	YeeE-TusA-DsrEFH type II	Sulfate, sulfite and S^0 reduced, thiosulfate not reduced, S^0 disproportionation	Friedrich et al. 1996, Finster 2008	GCF 900130015
Desulfomarina profundi KT2^T (DSM 111364^T)	YeeE-TusA-DsrEFH type II	Sulfate, sulfite and thiosulfate reduced	Hashimoto et al. 2021	GCF 019703855
Desulfopila aestuarii DSM 18488^T	YeeE-TusA-DsrEFH type II	Sulfate, thiosulfate and sulfite reduced	Suzuki et al. 2007b	GCF 900143695
Desulfoprunum benzoelyticum DSM 28570^T	YeeE-TusA-DsrEFH type II	Sulfate, thiosulfate and dimethyl sulfoxide reduced, S^0 and sulfite not reduced	Junghare and Schink 2015	GCF 014201505
Desulforhopalus singaporensis DSM 12130^T	YeeE-TusA-DsrEFH type II	Sulfate, sulfite and thiosulfate reduced, taurine fermented, sulfite disproportionation*	Lie et al. 1999, Kuever et al. 2015	GCF 900104445
Strain M19 (UBOCC-M-3423^T)	YeeE-TusA-DsrEFH type II	S^0 disproportionated	Hemon et al. 2025	JBMERE000000000
Desulfurivibrionaceae				
Desulfurivibrio alkaliphilus AHT 2^T (DSM 19089^T)	TusA-DsrEFH type II	Thiosulfate and S^0/polysulfide reduced, sulfate not reduced, sulfide oxidized, S^0 disproportionated	Sorokin, Tourova, Mussmann et al. 2008, Thorup et al. 2017; Chen et al. 2025	GCF 000092205
*Desulfurivibrio dismutans*AMeS2	TusA-DsrEFH type II	S^0 disproportionation	Sorokin et al. 2025	GCA_029210385
Thiovibrio frasassiensis RS19-109^T (DSM 115074^T)	YeeE-TusA-DsrEFH type II	Sulfate, thiosulfate and S^0 reduced; S^0 and thiosulfate disproportionated, sulfite not disproportionated, sulfide not oxidized	Aronson et al. 2023	GCF 029607905

Desulfobacteria				
Desulfobacterales				
Desulfobacteraceae				
Desulfocicer vacuolatum DSM 3385[T] *(previously Desulfobacterium vacuolatum)*	YeeE-TusA-DsrEFH type II	Sulfate reduced	Widdel 1988	GCF 900176365
Desulfospira joergensenii DSM 10085[T]	YeeE-TusA-DsrEFH type II	Sulfate, sulfur, thiosulfate and sulfite reduced	Finster et al. 1997	GCF 000420085
Desulfosarcinaceae				
Desulfatitalea tepidiphila S28bF[T] (DSM 23472[T])	DsrEFH type II	Sulfate and thiosulfate reduced	Higashioka et al. 2013	GCF 001293685
Desulfosarcina cetonica DSM 7267[T] *(previously Desulfobacterium centonicum)*	YeeE-TusA-DsrHFEE	Sulfate reduced	Galushka and Rozanova 1994	GCF 001311845
ETH-SRB1				
Desulfosarcina widdelii PP31[T] (DSM 103291[T])	YeeE-TusA-DsrEFH type II	Sulfate, thiosulfate and S^0 reduced, sulfite not reduced	Watanabe et al. 2017	GCF 009688965
Thermodesulfobacteria				
Thermodesulfobacteriales				
ST65				
Thermosulfuriphilus ammonigenes ST65[T] (DSM 102941[T])	YeeE-TusA-DsrEFH type II	S^0 oxidized, S^0, sulfite and thiosulfate disproportionated	Slobodkina et al. 2020	GCF 011207455
Thermodesulfatatoraceae				
Thermodesulfatator atlanticus DSM 21156[T] = AT1325[T]	YeeE-TusA-DsrEFH type II	Sulfate reduced, S^0 disproportionated; no growth on thiosulfate, sulfite	Alain et al. 2010 Allioux 2021	GCF 000421585
Thermodesulfatator autotrophicus S606[T] (DSM 101864[T])	TusA-DsrEFH type II	Sulfate reduced, thiosulfate, sulfite and S^0 not reduced	Lai et al. 2016	GCF 001642325
Thermodesulfatator indicus DSM 15286[T]	YeeE-TusA-DsrEFH type II	Sulfate reduced, S^0, thiosulfate, sulfite not reduced	Moussard et al. 2004	GCF 000217795
Thermodesulfobacteriaceae				
Caldimicrobium thiodismutans TF1[T] (DSM 29380[T])	YeeE-TusA-DsrEFH type II	S^0, thiosulfate and sulfite disproportionated, sulfate and thiosulfate not reduced	Kojima et al. 2016	GCF 001548275

		Thermosulfurimonas marina SU872[T] (DSM 104922[T])	YeeE-TusA-DsrEFH type II	S^0, thiosulfate and sulfite disproportionated, sulfate not reduced, S^0, thiosulfate and sulfite oxidized with nitrate, sulfide not oxidized	Frolova et al. 2018	GCF 012317585
		Thermosulfurimonas dismutans S95[T] (DSM 24515[T])	YeeE-TusA-DsrEFH type II	Thiosulfate, sulfite and S^0 disproportionated, no sulfate reduction	Slobodkin et al. 2012, Mardanov et al. 2016	GCF 001652585
		Thermosulfurimonas sp. F29	YeeE-TusA-DsrEFH type II	S^0 disproportionated, no sulfate reduction	Allioux et al. 2022	ASM1968873v1
**DSM-4660**						
Desulfatiglandales						
	Desulfatiglandaceae					
		Desulfatiglans anilini DSM 4660[T]	DsrK-DsrK-DsrEFH type II-TusA-YeeE	Sulfate and sulfite reduced, S^0 not reduced, dismutation of thiosulfate without growth	Schnell et al. 1989, Suzuki et al. 2014	GCF 000422285
Dissulfuribacteria						
Dissulfuribacterales						
	Dissulfuribacteraceae					
		Dissulfuribacter thermophilus S69[T] (DSM 25762[T])	YeeE-TusA-DsrEFH type II	Thiosulfate, sulfite and S^0 disproportionated, sulfate reduced	Slobodkin et al. 2013, Ailloux 2021	GCF 001687335
		Dissulfurirhabdus thermomarina SH388T	YeeE-TusA-DsrEFH type II	Sulfite and S^0 disproportionated, thiosulfate not dismutated, sulfite reduced, sulfate not reduced	Slobodkina et al. 2016	GCF 012979235
	Sh68					
		Dissulfurimicrobium hydrothermale Sh68[T] (JCM 19990[T])	YeeE-TusA-DsrEFH type II	S^0, thiosulfate and sulfite disproportionated, sulfate not reduced, reduces elemental sulfur	Slobodkin et al. 2016	GCF 022026155

Nitrospirota							
	Thermodesulfovibrionia						
		Thermodesulfovibrionales					
			Dissulfurispiraceae				
				Dissulfurispira thermophila T55J^T (DSM 110365^T)	YeeE-DsrEFH type II	S^0 and thiosulfate disproportionated	Umezawa et al. 2021	GCF 014701235

Desulforhopalus singaporensis DSM 12130^T may grow by disproportionation of sulfite but not thiosulfate, because it was reported that this bacterium grew with sulfite as a sole energy source and acetate as a carbon source but did not grow with thiosulfate and acetate (Lie et al. 1999, Kuever et al. 2015, Umezawa et al. 2020)

References

Alain, K., Postec, A., Grinsard, E., Lesongeur, F., Prieur, D., & Godfroy, A. (2010). *Thermodesulfatator atlanticus* sp. nov., a thermophilic, chemolithoautotrophic, sulfate-reducing bacterium isolated from a Mid-Atlantic Ridge hydrothermal vent. International Journal of Systematic and Evolutionary Microbiology, 60(Pt 1), 33-38. https://doi.org/10.1099/ijs.0.009449-0

Allioux M. (2021). Etudes Physiologiques et Multi-Omiques de métabolismes du Soufre présents Dans les écosystèmes Hydrothermaux: Physiological and Multi-Omics Studies of Microbial Sulfur Metabolisms Present in Hydrothermal Ecosystems, France: These de doctorat, Université de Bretagne Occidentale, Brest. https://theses.hal.science/tel-03789624v1

Allioux, M., Yvenou, S., Godfroy, A., Shao, Z., Jebbar, M., & Alain, K. (2022). Genome analysis of a new sulphur disproportionating species *Thermosulfurimonas* strain F29 and comparative genomics of sulfur-disproportionating bacteria from marine hydrothermal vents. *Microbial Genomics*, *8*(9), 000865

An, T. T., & Picardal, F. W. (2014). *Desulfocarbo indianensis* gen. nov., sp. nov., a benzoate-oxidizing, sulfate-reducing bacterium isolated from water extracted from a coal bed. International Journal of Systematic and Evolutionary Microbiology, 64(Pt 8), 2907-2914. https://doi.org/10.1099/ijs.0.064873-0

Chen, S.-C., Li, X.-M., Battisti, N., Guan, G., Montoya, M.A., Osvatic, J., Pjevac, P., Pollak, S., Richter, A., Schintlmeister, A., Wanek, W., Mussmann, M. & & Loy, A. (2025). Microbial iron oxide respiration coupled to sulfide oxidation. Nature. doi: 10.1038/s41586-025-09467-0.

Cross, K. L., Chirania, P., Xiong, W., Beall, C. J., Elkins, J. G., Giannone, R. J., Griffen, A. L., Guss, A. M., Hettich, R. L., Joshi, S. S., Mokrzan, E. M., Martin, R. K., Zhulin, I. B., Leys, E. J., Podar, M., Dewhirst, F. E., & Dominguez Bello, M. G. (2018). Insights into the evolution of host association through the isolation and characterization of a novel human periodontal pathobiont, *Desulfobulbus oralis*. Mbio, 9(2). https://doi.org/10.1128/mBio.02061-17

El Houari, A., Ranchou-Peyruse, M., Ranchou-Peyruse, A., Dakdaki, A., Guignard, M., Idouhammou, L., Bennisse, R., Bouterfass, R., Guyoneaud, R., & Qatibi, A. I. (2017). *Desulfobulbus oligotrophicus* sp. nov., a sulfate-reducing and propionate-oxidizing bacterium isolated from a municipal anaerobic sewage sludge digester. International Journal of Systematic and Evolutionary Microbiology, 67(2), 275-281. https://doi.org/10.1099/ijsem.0.001615

Finster, K., Liesack, W., & Thamdrup, B. (1998). Elemental sulfur and thiosulfate disproportionation by *Desulfocapsa sulfoexigens* sp. nov., a new anaerobic bacterium isolated from marine surface sediment. *Applied and Environmental Microbiology, 64* , 119-125.

Finster, K., Liesack, W., & Tindall, B. J. (1997). *Desulfospira joergensenii*, gen. nov., sp. nov., a new sulfate-reducing bacterium isolated from marine surface sediment. Systematic and Applied Microbiology, 20(2), 201-208. https://doi.org/10.1016/s0723-2020(97)80066-5

Finster, K. (2008). Microbiological disproportionation of inorganic sulfur compounds. Journal of Sulfur Chemistry, 29, 281-292. https://doi.org/10.1080/17415990802105770

Friedrich, M., Springer, N., Ludwig, W., & Schink, B. (1996). Phylogenetic positions of *Desulfofustis glycolicus* gen. nov., sp. nov. and *Syntrophobotulus glycolicus* gen. nov., sp. nov., two new strict anaerobes growing with glycolic acid. International Journal of Systematic Bacteriology, 46(4), 1065-1069. https://doi.org/10.1099/00207713-46-4-1065

Frolova, A. A., Slobodkina, G. B., Baslerov, R. V., Novikov, A. A., Bonch-Osmolovskaya, E. A., & Slobodkin, A. I. (2018). *Thermosulfurimonas marina* sp. nov., an autotrophic sulfur-disproportionating and nitrate-reducing bacterium isolated from a shallow-sea hydrothermal vent. Microbiology, 87(4), 502-507. https://doi.org/10.1134/s0026261718040082

Galushko, A., & Kuever, J. (2019). *Desulforhopalus Bergey's manual of systematics of archaea and bacteria* (pp. 1-5).

Hashimoto, Y., Shimamura, S., Tame, A., Sawayama, S., Miyazaki, J., Takai, K., & Nakagawa, S. (2022). Physiological and comparative proteomic characterization of *Desulfolithobacter dissulfuricans* gen. nov., sp. nov., a novel mesophilic, sulfur-disproportionating chemolithoautotroph from a deep-sea hydrothermal vent. Frontiers in Microbiology, 13. https://doi.org/10.3389/fmicb.2022.1042116

Hashimoto, Y., Tame, A., Sawayama, S., Miyazaki, J., Takai, K., & Nakagawa, S. (2021). *Desulfomarina profundi* gen. nov., sp. nov., a novel mesophilic, hydrogen-oxidizing, sulphate-reducing chemolithoautotroph isolated from a deep-sea hydrothermal vent chimney. International Journal of Systematic and Evolutionary Microbiology, 71(11). https://doi.org/10.1099/ijsem.0.005083

Hemon, M., Novák, L., Allioux, M., Ailliot, L., Vince, E., & Alain, K. (2025). Draft genome sequence of Desulfobacterota strain M19, a mesophilic sulfur-disproportionating bacterium from a deep-sea hydrothermal vent on the Mid-Atlantic Ridge. *Microbiology Resource Announcements*, *14*(7), e00295-25.

Higashioka, Y., Kojima, H., Watanabe, M., & Fukui, M. (2013). *Desulfatitalea tepidiphila* gen. nov., sp. nov., a sulfate-reducing bacterium isolated from tidal flat sediment. International Journal of Systematic and Evolutionary Microbiology, 63(Pt 2), 761-765. https://doi.org/10.1099/ijs.0.043356-0

Kojima, H., Umezawa, K., & Fukui, M. (2016). *Caldimicrobium thiodismutans* sp. nov., a sulfur-disproportionating bacterium isolated from a hot spring, and emended description of the genus *Caldimicrobium*. International Journal of Systematic and Evolutionary Microbiology, 66(4), 1828-1831. https://doi.org/10.1099/ijsem.0.000947

Kuever, J., Rainey, F. A., Widdel, F. (2015). Desulforhopalus. Bergey's Manual of Systematics of Archaea and Bacteria, 1-4. https://doi.org/10.1002/9781118960608.gbm01026

Lai, Q., Cao, J., Dupont, S., Shao, Z., Jebbar, M., & Alain, K. (2016). *Thermodesulfatator autotrophicus* sp. nov., a thermophilic sulfate-reducing bacterium from the Indian Ocean. International Journal of Systematic and Evolutionary Microbiology, 66(10), 3978-3982. https://doi.org/10.1099/ijsem.0.001297

Lie, T. J., Clawson, M. L., Godchaux, W., & Leadbetter, E. R. (1999). Sulfidogenesis from 2-aminoethanesulfonate (taurine) fermentation by a morphologically unusual sulfate-reducing bacterium, *Desulforhopalus singaporensis* sp. nov. Applied and Environmental Microbiology, 65(8), 3328-3334. https://doi.org/10.1128/AEM.65.8.3328-3334.1999

Lien, T., Madsen, M., Steen, I. H., & Gjerdevik, K. (1998). *Desulfobulbus rhabdoformis* sp. nov., a sulfate reducer from a water-oil separation system. International Journal of Systematic Bacteriology, 48 Pt 2, 469-474. https://doi.org/10.1099/00207713-48-2-469

Mardanov, A. V., Beletsky, A. V., Kadnikov, V. V., Slobodkin, A. I., & Ravin, N. V. (2016). Genome analysis of *Thermosulfurimonas dismutans*, the first thermophilic sulfur-disproportionating bacterium of the phylum Thermodesulfobacteria. Frontiers in Microbiology, 7, 950. https://doi.org/10.3389/fmicb.2016.00950

Moussard, H., L'Haridon, S., Tindall, B. J., Banta, A., Schumann, P., Stackebrandt, E., Reysenbach, A. L., & Jeanthon, C. (2004). *Thermodesulfatator indicus* gen.

nov., sp. nov., a novel thermophilic chemolithoautotrophic sulfate-reducing bacterium isolated from the Central Indian Ridge. International Journal of Systematic and Evolutionary Microbiology, 54(Pt 1), 227-233. https://doi.org/10.1099/ijs.0.02669-0

Pagani, I., Lapidus, A., Nolan, M., Lucas, S., Hammon, N., Deshpande, S., Cheng, J. F., Chertkov, O., Davenport, K., Tapia, R., Han, C., Goodwin, L., Pitluck, S., Liolios, K., Mavromatis, K., Ivanova, N., Mikhailova, N., Pati, A., Chen, A., Palaniappan, K., Land, M., Hauser, L., Chang, Y. J., Jeffries, C. D., Detter, J. C., Brambilla, E., Kannan, K. P., Djao, O. D., Rohde, M., Pukall, R., Spring, S., Goker, M., Sikorski, J., Woyke, T., Bristow, J., Eisen, J. A., Markowitz, V., Hugenholtz, P., Kyrpides, N. C., & Klenk, H. P. (2011). Complete genome sequence of *Desulfobulbus propionicus* type strain (1pr3). Stand Genomic Sci, 4(1), 100-110. https://doi.org/10.4056/sigs.1613929

Samain, E., Dubourguier, H. C., & Albagnac, G. (1984). Isolation and characterization of *Desulfobulbus elongatus* sp. nov. from a mesophilic industrial digester. Systematic and Applied Microbiology, 5(3), 391-401. https://doi.org/10.1016/s0723-2020(84)80040-5

Sass, A., Rütters, H., Cypionka, H., & Sass, H. (2002). *Desulfobulbus mediterraneus* sp. nov., a sulfate-reducing bacterium growing on mono- and disaccharides. Archives of Microbiology, 177(6), 468-474. https://doi.org/10.1007/s00203-002-0415-5

Schnell, S., Bak, F., & Pfennig, N. (1989). Anaerobic degradation of aniline and dihydroxybenzenes by newly isolated sulfate-reducing bacteria and description of *Desulfobacterium anilini*. Archives of Microbiology, 152(6), 556-563. https://doi.org/Doi 10.1007/Bf00425486

Slobodkin, A. I., Reysenbach, A. L., Slobodkina, G. B., Baslerov, R. V., Kostrikina, N. A., Wagner, I. D., & Bonch-Osmolovskaya, E. A. (2012). *Thermosulfurimonas dismutans* gen. nov., sp. nov., an extremely thermophilic sulfur-disproportionating bacterium from a deep-sea hydrothermal vent. International Journal of Systematic and Evolutionary Microbiology, 62(Pt 11), 2565-2571. https://doi.org/10.1099/ijs.0.034397-0

Slobodkin, A. I., Reysenbach, A. L., Slobodkina, G. B., Kolganova, T. V., Kostrikina, N. A., & Bonch-Osmolovskaya, E. A. (2013). *Dissulfuribacter thermophilus* gen. nov., sp. nov., a thermophilic, autotrophic, sulfur-disproportionating, deeply branching deltaproteobacterium from a deep-sea hydrothermal vent. International Journal of Systematic and Evolutionary Microbiology, 63(Pt 6), 1967-1971. https://doi.org/10.1099/ijs.0.046938-0

Slobodkin, A. I., Slobodkina, G. B., Panteleeva, A. N., Chernyh, N. A., Novikov, A. A., & Bonch-Osmolovskaya, E. A. (2016). *Dissulfurimicrobium hydrothermale* gen. nov., sp. nov., a thermophilic, autotrophic, sulfur-disproportionating deltaproteobacterium isolated from a hydrothermal pond. International Journal of Systematic and Evolutionary Microbiology, 66(2), 1022-1026. https://doi.org/10.1099/ijsem.0.000828

Slobodkina, G., Allioux, M., Merkel, A., Alain, K., Jebbar, M., & Slobodkin, A. (2020). Genome analysis of *Thermosulfuriphilus ammonigenes* ST65(T), an anaerobic thermophilic chemolithoautotrophic bacterium isolated from a deep-sea hydrothermal vent. Marine Genomics, 54, 100786. https://doi.org/10.1016/j.margen.2020.100786

Slobodkina, G. B., Kolganova, T. V., Kopitsyn, D. S., Viryasov, M. B., Bonch-Osmolovskaya, E. A., & Slobodkin, A. I. (2016). *Dissulfurirhabdus thermomarina* gen. nov., sp. nov., a thermophilic, autotrophic, sulfite-reducing and disproportionating deltaproteobacterium isolated from a shallow-sea hydrothermal vent. International Journal of Systematic and Evolutionary Microbiology, 66(7), 2515-2519. https://doi.org/10.1099/ijsem.0.001083

Sorokin, D. Y., Merkel, A. Y., Ziganshin, R. H., & Kublanov, I. V. (2025). Growth physiology, genomics, and proteomics of *Desulfurivibrio dismutans* sp. nov., an obligately chemolithoautotrophic, sulfur disproportionating and ammonifying haloalkaliphile from soda lakes. *Frontiers in Microbiology*, *16*, 1590477.

Sorokin, D. Y., Tourova, T. P., Henstra, A. M., Stams, A. J. M., Galinski, E. A., & Muyzer, G. (2008). Sulfidogenesis under extremely haloalkaline conditions by *Desulfonatronospira thiodismutans* gen. nov., sp. nov., and *Desulfonatronospira delicata* sp. nov. - a novel lineage of Deltaproteobacteria from hypersaline soda lakes. *Microbiology, 154* , 1444-1453.

Sorokin, D. Y., Tourova, T. P., Kolganova, T. V., Detkova, E. N., Galinski, E. A., & Muyzer, G. (2011). Culturable diversity of lithotrophic haloalkaliphilic sulfate-reducing bacteria in soda lakes and the description of *Desulfonatronum thioautotrophicum* sp. nov., *Desulfonatronum thiosulfatophilum* sp. nov., *Desulfonatronovibrio thiodismutans* sp. nov., and *Desulfonatronovibrio magnus* sp. nov. Extremophiles, 15(3), 391-401. https://doi.org/10.1007/s00792-011-

0370-7
Sorokin, D. Y., Tourova, T. P., Mussmann, M., & Muyzer, G. (2008). *Dethiobacter alkaliphilus* gen. nov. sp. nov., and *Desulfurivibrio alkaliphilus* gen. nov. sp. nov.: two novel representatives of reductive sulfur cycle from soda lakes. *Extremophiles, 12* (3), 431-439.
Sorokin, D. Y., Tourova, T. P., Panteleeva, A. N., & Muyzer, G. (2012). *Desulfonatronobacter acidivorans* gen. nov., sp. nov. and *Desulfobulbus alkaliphilus* sp. nov., haloalkaliphilic heterotrophic sulfate-reducing bacteria from soda lakes. Int J Syst Evol Microbiol, 62(Pt 9), 2107-2113. https://doi.org/10.1099/ijs.0.029777-0
Suzuki, D., Li, Z., Cui, X., Zhang, C., & Katayama, A. (2014). Reclassification of *Desulfobacterium anilini* as *Desulfatiglans anilini* comb. nov. within *Desulfatiglans* gen. nov., and description of a 4-chlorophenol-degrading sulfate-reducing bacterium, *Desulfatiglans parachlorophenolica* sp. nov. International Journal of Systematic and Evolutionary Microbiology, 64(Pt 9), 3081-3086. https://doi.org/10.1099/ijs.0.064360-0
Suzuki, D., Ueki, A., Amaishi, A., & Ueki, K. (2007a). *Desulfobulbus japonicus* sp. nov., a novel Gram-negative propionate-oxidizing, sulfate-reducing bacterium isolated from an estuarine sediment in Japan. International Journal of Systematic and Evolutionary Microbiology, 57(Pt 4), 849-855. https://doi.org/10.1099/ijs.0.64855-0
Suzuki, D., Ueki, A., Amaishi, A., & Ueki, K. (2007b). *Desulfopila aestuarii* gen. nov., sp. nov., a Gram-negative, rod-like, sulfate-reducing bacterium isolated from an estuarine sediment in Japan. International Journal of Systematic and Evolutionary Microbiology, 57(Pt 3), 520-526. https://doi.org/10.1099/ijs.0.64600-0
Thorup, C., Schramm, A., Findlay, A.J., Finster, K.W. & Schreiber, L. (2017) Disguised as a sulfate reducer: Growth of the deltaproteobacterium *Desulfurivibrio alkaliphilus* by Sulfide Oxidation with Nitrate. mBio 8(4) 1-9. doi: 10.1128/mBio.00671-17
Umezawa, K., Kojima, H., Kato, Y., & Fukui, M. (2020). Disproportionation of inorganic sulfur compounds by a novel autotrophic bacterium belonging to Nitrospirota. Systematic and Applied Microbiology, 43(5), 126110. https://doi.org/10.1016/j.syapm.2020.126110
Umezawa, K., Kojima, H., Kato, Y., & Fukui, M. (2021). *Dissulfurispira thermophila* gen. nov., sp. nov., a thermophilic chemolithoautotroph growing by sulfur disproportionation, and proposal of novel taxa in the phylum Nitrospirota to reclassify the genus Thermodesulfovibrio. Systematic and Applied Microbiology, 44(2), 126184. https://doi.org/10.1016/j.syapm.2021.126184
Watanabe, M., Higashioka, Y., Kojima, H., & Fukui, M. (2017). *Desulfosarcina widdelii* sp. nov. and *Desulfosarcina alkanivorans* sp. nov., hydrocarbon-degrading sulfate-reducing bacteria isolated from marine sediment and emended description of the genus Desulfosarcina. International Journal of Systematic and Evolutionary Microbiology, 67(8), 2994-2997. https://doi.org/10.1099/ijsem.0.002062
Watanabe, M., Takahashi, A., Kojima, H., Miyata, N., & Fukui, M. (2022). *Desulfofustis limnaeus* sp. nov., a freshwater sulfate-reducing bacterium isolated from marsh soil. Archives of Microbiology, 204(10). https://doi.org/10.1007/s00203-022-03261-6
Widdel, F. (1988). Microbiology and ecology of sulfate- and sulfur-reducing bacteria. In J. B. Zehnder (Ed.), *Biology of anaerobic microorganisms* (pp. 469-585). New York: John Wiley & Sons.
Widdel, F., & Bak, F. (1992). Gram-negative mesophilic sulfate-reducing bacteria. In A. Balows, H. G. Trüper, M. Dworkin, W. Harder, & K. H. Schleifer (Eds.), *The prokaryotes* (2 ed., pp. 3352-3378). New York: Springer.
Zhilina, T. N., Zavarzin, G. A., Rainey, F. A., Pikuta, E. N., Osipov, G. A., & Kostrikina, N. A. (1997). *Desulfonatronovibrio hydrogenovorans* gen. nov., sp. nov., an alkaliphilic, sulfate-reducing bacterium. International Journal of Systematic Bacteriology, 47(1), 144-149. https://doi.org/10.1099/00207713-47-

Table S6: Analysis of known sulfur disproportionators

Sequenced genomes of 63 species known as sulfur disproportionators that are available as pure cultures were analyzed for the presence of the *dsrEFH* type II and compared to the organisms' capacity for sulfur disproportionation. **+** = yes, **-** = no, NR = not reported/not tested.

Known sulfur disproportionators			***dsrEFH* type II**	**Disproportionation of**			**Reference**
**Phylum**	**Order**	**Species**		**S^0**	**$S_2O_3^{2-}$**	**SO_3^{2-}**	
Bacillota	*Clostridiales*	*Desulfofundulus salinum* 435	-	NR	+	NR	Nazina et al. 2005
		Desulfofundulus thermobenzoicum TSB[T]	-	NR	+	NR	Jackson & McInerney 2000
		Desulfotomaculum nigrificans DSM 574[T]	-	NR	-	+	Krämer & Cypionka 1989
		Dethiobacter alkaliphilus AHT 1[T]	-	+	NR	NR	Poser et al. 2013
Campylobacterota	*Campylobacterales*	*Sulfurimonas hydrogeniphila* NW10[T]	-	-	+	-	Wang et al. 2022
		Sulfurimonas marina B 2[T]	-	-	+	NR	Wang et al. 2022
		Sulfurimonas sp. HSL1-2	-	+	+	NR	Wang et al. 2022
		Sulfurimonas sp. HSL1-6	-	+	+	NR	Wang et al. 2022
		Sulfurimonas sp. HSL3-1	-	+	+	NR	Wang et al. 2022
		Sulfurimonas sp. HSL3-2	-	+	+	NR	Wang et al. 2022
		Sulfurimonas sp. HSL3-7	-	+	+	NR	Wang et al. 2022
		Sulfurimonas sp. ST-25	-	+	+	NR	Wang et al. 2022
		Sulfurimonas sp. ST-27	-	+	+	NR	Wang et al. 2022
		Sulfurimonas sp. NWX367	-	+	+	NR	Wang et al. 2022
		Sulfurimonas sp. NWX79	-	+	+	NR	Wang et al. 2022
		Sulfurovum riftiae 1812E[T]	-	+	+	-	Wang et al. 2022
		Sulfurovum sp. HSL1-3 ST1-3	-	+	+	NR	Wang et al. 2022
		Sulfurovum sp. ST-21	-	+	+	NR	Wang et al. 2022
		Sulfurovum sp. ST-29	-	+	+	NR	Wang et al. 2022
Pseudomonadota	*Bacillales*	*Exiguobacterium sp.*	-	+	-	-	Wu et al. 2025
	Enterobacteriales	*Pantoea agglomerans* SP1	-	+	NR	NR	Obraztsova et al. 2010
Desulfobacterota	*Desulfobulbales*	*Desulfobulbus propionicus* 1pr3[T]	+	+	-	-	Lovley & Phillips, 1994
		Desulfobulbus oligotrophicus Prop6[T]	+	-	-	+	El Houari et al. 2017
		Desulfolithobacter dissulfuricans GF1[T]	+	+	+	-	Hashimoto et al. 2022
		Desulfocapsa sulfexigens SB164P1[T]	+	+	+	+	Finster et al. 1998
		Desulfocapsa thiozymogenes Bra2[T]	-	+	+	+	Janssen et al. 1996
		Desulfofustis glycolicus PerGlys[T]	+	+	NR	NR	Finster 2008
		Desulforhopalus singaporensis T1[T]	+	NR	NR	+	Lie et al. 1999
		Desulfurivibrio alkaliphilus AHT 2[T]	+	+	NR	NR	Thorup et al. 2017

**Desulfobacterota**		*Desulfurivibrio dismutans* AMeS2[T]	+	+	-	-	Sorokin et al. 2025
		Desulfocapsaceae strain M19	+	+	NR	NR	Hemon et al. 2025
		Thiovibrio frasassiensis RS19-109[T]	+	+	+	-	Aronson et al. 2023
	Desulfovibrionales	*Desulfonatronospira delicata* AHT 6[T]	-	-	+	NR	Sorokin et al. 2008
		Desulfonatronospira thiodismutans ASO3-1[T]	+	-	+	+	Sorokin et al. 2008
		Desulfonatronovibrio magnus AHT22[T]	+	-	+	+	Sorokin et al. 2011
		Desulfonatronovibrio thiodismutans AHT9[T]	-	-	+	+	Sorokin et al. 2011
		Desulfonatronovibrio hydrogenovorans Z-7935[T]	+	-	+	+	Zhilina et al. 1997
		Desulfonatronum lacustre Z-7951[T]	-	-	+	NR	Pikuta et al. 1998
		Desulfonatronum thioautotrophicum ASO4-1[T]	+	-	+	+	Sorokin et al. 2011
		Desulfonatronum thiodismutans MLF1[T]	-	-	+	+	Pikuta et al. 2003
		Desulfonatronum thiosulfatophilum ASO4-2[T]	-	-	+	+	Sorokin et al. 2011
		Desulfonatronum parangueonense PAR180[T]	-	-	+	+	Perez et al. 2017
		Salidesulfovibrio brasiliensis LVform1[T]	-	NR	+	NR	Warthmann et al. 2005
		Desulfovibrio aminophilus ALA-3[T]	-	NR	+	+	Baena et al. 1998
		Desulfovibrio desulfuricans CSN	-	NR	-	+	Krämer & Cypionka 1989
		Desulfovibrio oxyclinae P1B[T]	-	NR	+	+	Krekeler et al. 1997
		Desulfolutivibrio sulfodismutans ThAc01[T]	-	-	+	+	Bak & Pfennig 1987
		Humidesulfovibrio mexicanus Lup1[T]	-	NR	+	-	Hernandez-Eugenio et al. 2000
		Paucidesulfovibrio longus DSM 6739[T]	-	NR	-	+	Magot et al. 1992
	Desulfurellales	*Desulfurella amilsii* TR1[T]	-	+	NR	NR	Florentino et al. 2016
	Desulfomonilales	*Desulfomonile tiedje* DCB-1[T]	-	NR	+	NR	Mohn & Tiedje 1990
	Desulfatiglandales	*Desulfatiglans anilini* ATCC 49792[T]	+	NR	+	NR	Schnell et al. 1989
	Dissulfuribacterales	*Dissulfuribacter thermophilus* S69[T]	+	+	+	+	Slobodkin et al. 2013
		Dissulfurimicrobium hydrothermale Sh68[T]	+	+	+	+	Slobodkin et al. 2016
		Dissulfurirhabdus thermomarina SH388[T]	+	+	-	+	Slobodkina et al. 2016
	Thermodesulfobacteriales	*Caldimicrobium thiodismutans* TF1[T]	+	+	+	+	Kojima et al. 2016
		Caldimicrobium rimae FM8	-	+	NR	NR	Merkel et al. 2017
		Thermodesulfatator atlanticus AT 1325[T]	+	+	-	-	Allioux 2021
		Thermosulfurimonas dismutans S95[T]	+	+	+	+	Mardanov et al. 2016
		Thermosulfurimonas marina S872[T]	+	+	+	+	Frolova et al. 2018
		Thermosulfurimonas sp. F29	+	+	+	-	Allioux et al. 2022
		Thermosulfuriphilus ammonigenes ST65[T]	+	+	+	+	Slobodkina et al. 2020
Nitrospirota	*Thermodesulfovibrionales*	*Dissulfurispira thermophila* T55J[T]	+	+	+	-	Umezawa et al. 2021

References

Allioux M. (2021). Etudes Physiologiques et Multi-Omiques de métabolismes du Soufre présents Dans les écosystèmes Hydrothermaux: Physiological and Multi-Omics Studies of Microbial Sulfur Metabolisms Present in Hydrothermal Ecosystems, France: These de doctorat, Université de Bretagne Occidentale, Brest.

Allioux, M., Yvenou, S., Godfroy, A., Shao, Z., Jebbar, M., & Alain, K. (2022). Genome analysis of a new sulphur disproportionating species *Thermosulfurimonas* strain F29 and comparative genomics of sulfur-disproportionating bacteria from marine hydrothermal vents. *Microbial Genomics*, *8*(9), 000865.

Aronson, H. S., Thomas, C., Bhattacharyya, M. K., Eckstein, S. R., Jensen, S. R., Barco, R. A., Macalady, J. L. & Amend, J. P. (2023). *Thiovibrio frasassiensis* gen. nov., sp. nov., an autotrophic, elemental sulphur disproportionating bacterium isolated from sulphidic karst sediment, and proposal of Thiovibrionaceae fam. nov. *International journal of systematic and evolutionary microbiology*, *73*(8), 006003.

Baena, S., Fardeau, M. L., Labat, M., Ollivier, B., Garcia, J. L., & Patel, B. K. C. (1998). *Desulfovibrio aminophilus* sp. nov., a novel amino acid degrading and sulfate reducing bacterium from an anaerobic dairy wastewater lagoon. *Systematic and applied microbiology*, *21*(4), 498-504.

Bak, F., & Pfennig, N. (1987). Chemolithotrophic growth of *Desulfovibrio sulfodismutans* sp. nov. by disproportionation of inorganic sulfur compounds. *Archives of Microbiology*, *147*(2), 184-189.

El Houari, A., Ranchou-Peyruse, M., Ranchou-Peyruse, A., Dakdaki, A., Guignard, M., Idouhammou, L., Bennisse, R., Bouterfass, R., Guypneaud, R. & Qatibi, A. I. (2017). *Desulfobulbus oligotrophicus* sp. nov., a sulfate-reducing and propionate-oxidizing bacterium isolated from a municipal anaerobic sewage sludge digester. *International journal of systematic and evolutionary microbiology*, *67*(2), 275-281.

Finster, K., Liesack, W., & Thamdrup, B. O. (1998). Elemental sulfur and thiosulfate disproportionation by *Desulfocapsa sulfoexigens* sp. nov., a new anaerobic bacterium isolated from marine surface sediment. *Applied and environmental microbiology*, *64*(1), 119-125.

Finster, K. (2008). Microbiological disproportionation of inorganic sulfur compounds. *Journal of Sulfur Chemistry*, *29*(3-4), 281-292.

Florentino, A. P., Brienza, C., Stams, A. J., & Sanchez-Andrea, I. (2016). *Desulfurella amilsii* sp. nov., a novel acidotolerant sulfur-respiring bacterium isolated from acidic river sediments. *International Journal of Systematic and Evolutionary Microbiology*, *66*(3), 1249-1253.

Frolova, A. A., Slobodkina, G. B., Baslerov, R. V., Novikov, A. A., Bonch-Osmolovskaya, E. A., & Slobodkin, A. I. (2018). *Thermosulfurimonas marina* sp. nov., an autotrophic sulfur-disproportionating and nitrate-reducing bacterium isolated from a shallow-sea hydrothermal vent. *Microbiology*, *87*(4), 502-507.

Hashimoto, Y., Shimamura, S., Tame, A., Sawayama, S., Miyazaki, J., Takai, K., & Nakagawa, S. (2022). Physiological and comparative proteomic characterization of *Desulfolithobacter dissulfuricans* gen. nov., sp. nov., a novel mesophilic, sulfur-disproportionating chemolithoautotroph from a deep-sea hydrothermal vent. *Frontiers in Microbiology*, *13*, 1042116.

Hemon, M., Novák, L., Allioux, M., Ailliot, L., Vince, E., & Alain, K. (2025). Draft genome sequence of Desulfobacterota strain M19, a mesophilic sulfur-disproportionating bacterium from a deep-sea hydrothermal vent on the Mid-Atlantic Ridge. *Microbiology Resource Announcements*, *14*(7), e00295-25.

Hernandez-Eugenio, G., Fardeau, M.-L., Patel, B. K. C., Macarie, H., Garcia, J.-L. & Ollivier, B. (2000). *Desulfovibrio mexicanus* sp. nov., a Sulfate-reducing bacterium isolated from an Upflow anaerobic sludge blanket (UASB) reactor treating cheese wastewaters. Anaerobe 2000;6:305–12.

Jackson, B. E., & McInerney, M. J. (2000). Thiosulfate disproportionation by *Desulfotomaculum thermobenzoicum*. *Applied and Environmental Microbiology*, *66*(8), 3650-3653.

Janssen, P. H., Schuhmann, A., Bak, F., & Liesack, W. (1996). Disproportionation of inorganic sulfur compounds by the sulfate-reducing bacterium *Desulfocapsa thiozymogenes* gen. nov., sp. nov. *Archives of Microbiology*, *166*(3), 184-192.

Kojima, H., Umezawa, K., & Fukui, M. (2016). *Caldimicrobium thiodismutans* sp. nov., a sulfur-disproportionating bacterium isolated from a hot spring, and emended description of the genus *Caldimicrobium*. *International journal of systematic and evolutionary microbiology*, *66*(4), 1828-1831.

Krämer, M., & Cypionka, H. (1989). Sulfate formation via ATP sulfurylase in thiosulfate-and sulfite-disproportionating bacteria. *Archives of microbiology*, *151*(3), 232-237.

Krekeler, D., Sigalevich, P., Teske, A., Cypionka, H., & Cohen, Y. (1997). A sulfate-reducing bacterium from the oxic layer of a microbial mat from Solar Lake (Sinai), *Desulfovibrio oxyclinae* sp. nov. *Archives of microbiology*, *167*(6), 369-375.

Lie, T. J., Clawson, M. L., Godchaux, W., & Leadbetter, E. R. (1999). Sulfidogenesis from 2-aminoethanesulfonate (taurine) fermentation by a morphologically unusual sulfate-reducing bacterium, *Desulforhopalus singaporensis* sp. nov. *Applied and environmental Microbiology*, *65*(8), 3328-3334.

Lovley, D. R., & Phillips, E. J. (1994). Novel processes for anaerobic sulfate production from elemental sulfur by sulfate-reducing bacteria. *Applied and Environmental Microbiology*, *60*(7), 2394-2399.

Magot, M., Caumette, P., Desperrier, J. M., Matheron, R., Dauga, C., Grimont, F., & Carreau, L. (1992). *Desulfovibrio longus* sp. nov., a sulfate-reducing bacterium isolated from an oil-producing well. *International Journal of Systematic and Evolutionary Microbiology*, *42*(3), 398-402.

Mardanov, A. V., Beletsky, A. V., Kadnikov, V. V., Slobodkin, A. I., & Ravin, N. V. (2016). Genome analysis of *Thermosulfurimonas dismutans*, the first thermophilic sulfur-disproportionating bacterium of the phylum Thermodesulfobacteria. *Frontiers in Microbiology*, *7*, 208689.

Merkel, A. Y., Pimenov, N. V., Rusanov, I. I., Slobodkin, A. I., Slobodkina, G. B., Tarnovetckii, I. Y., Frolov, E. N., Dubin, A. V., Perevalova, A. A. & Bonch-Osmolovskaya, E. A. (2017). Microbial diversity and autotrophic activity in Kamchatka hot springs. *Extremophiles*, *21*(2), 307-317.

Mohn, W. W., & Tiedje, J. M. (1990). Catabolic thiosulfate disproportionation and carbon dioxide reduction in strain DCB-1, a reductively dechlorinating anaerobe. *Journal of bacteriology*, *172*(4), 2065-2070.

Nazina, T. N., Rozanova, E. P., Belyakova, E. V., Lysenko, A. M., Poltaraus, A. B., Tourova, T. P., Osipov, G. A. & Belyaev, S. S. (2005). Description of "*Desulfotomaculum nigrificans* subsp. *salinus*" as a New Species, *Desulfotomaculum salinum* sp. nov. *Microbiology*, *74*(5), 567-574.

Obraztsova, A. Y., Francis, C. A., & Tebo, B. M. (2002). Sulfur disproportionation by the facultative anaerobe *Pantoea agglomerans* SP1 as a mechanism for chromium (VI) reduction. *Geomicrobiology Journal*, *19*(1), 121-132.

Perez Bernal, M. F., Souza Brito, E. M., Bartoli, M., Aubé, J., Fardeau, M. L., Cuevas Rodriguez, G., Ollivier, B., Guyoneaud, R. & Hirschler-Réa, A. (2017). *Desulfonatronum parangueonense* sp. nov., a sulfate-reducing bacterium isolated from sediment of an alkaline crater lake. *International Journal of Systematic and Evolutionary Microbiology*, *67*(12), 4999-5005.

Pikuta, E. V., Zhilina, T. N., Zavarzin, G. A., Kostrikina, N. A., Osipov, G. A., & Rainey, F. A. (1998). *Desulfonatronum lacustre* gen. nov., sp. nov.: a new alkaliphilic sulfate-reducing bacterium utilizing ethanol. *Microbiology+*, *67*(1), 105-113.

Pikuta, E. V., Hoover, R. B., Bej, A. K., Marsic, D., Whitman, W. B., Cleland, D., & Krader, P. (2003). *Desulfonatronum thiodismutans* sp. nov., a novel alkaliphilic, sulfate-reducing bacterium capable of lithoautotrophic growth. *International Journal of Systematic and Evolutionary Microbiology*, *53*(5), 1327-1332.

Poser, A., Lohmayer, R., Vogt, C., Knoeller, K., Planer-Friedrich, B., Sorokin, D., Richnow, H.-H. & Finster, K. (2013). Disproportionation of elemental sulfur by haloalkaliphilic bacteria from soda lakes. *Extremophiles*, *17*(6), 1003-1012.

Schnell, S., Bak, F., & Pfennig, N. (1989). Anaerobic degradation of aniline and dihydroxybenzenes by newly isolated sulfate-reducing bacteria and description of *Desulfobacterium anilini*. *Archives of Microbiology*, *152*(6), 556-563.

Slobodkin, A. I., Reysenbach, A. L., Slobodkina, G. B., Kolganova, T. V., Kostrikina, N. A., & Bonch-Osmolovskaya, E. A. (2013). *Dissulfuribacter thermophilus* gen. nov., sp. nov., a thermophilic, autotrophic, sulfur-disproportionating, deeply branching deltaproteobacterium from a deep-sea hydrothermal vent. *International journal of systematic and evolutionary microbiology*, *63*(Pt_6), 1967-1971.

Slobodkin, A. I., Slobodkina, G. B., Panteleeva, A. N., Chernyh, N. A., Novikov, A. A., & Bonch-Osmolovskaya, E. A. (2016). *Dissulfurimicrobium hydrothermale* gen. nov., sp. nov., a thermophilic, autotrophic, sulfur-disproportionating deltaproteobacterium isolated from a hydrothermal pond. *International Journal of Systematic and Evolutionary Microbiology*, *66*(2), 1022-1026.

Slobodkina, G. B., Kolganova, T. V., Kopitsyn, D. S., Viryasov, M. B., Bonch-Osmolovskaya, E. A., & Slobodkin, A. I. (2016). *Dissulfurirhabdus thermomarina* gen. nov., sp. nov., a thermophilic, autotrophic, sulfite-reducing and disproportionating deltaproteobacterium isolated from a shallow-sea hydrothermal vent. *International journal of systematic and evolutionary microbiology*, *66*(7), 2515-2519.

Slobodkina, G., Allioux, M., Merkel, A., Alain, K., Jebbar, M., & Slobodkin, A. (2020). Genome analysis of *Thermosulfuriphilus ammonigenes* ST65T, an anaerobic thermophilic chemolithoautotrophic bacterium isolated from a deep-sea hydrothermal vent. *Marine genomics*, *54*, 100786.

Sorokin, D. Y., Tourova, T. P., Henstra, A. M., Stams, A. J., Galinski, E. A., & Muyzer, G. (2008). Sulfidogenesis under extremely haloalkaline conditions by *Desulfonatronospira thiodismutans* gen. nov., sp. nov., and *Desulfonatronospira delicata* sp. nov.–a novel lineage of Deltaproteobacteria from hypersaline soda lakes. *Microbiology*, *154*(5), 1444-1453.

Sorokin, D. Y., Tourova, T. P., Kolganova, T. V., Detkova, E. N., Galinski, E. A., & Muyzer, G. (2011). Culturable diversity of lithotrophic haloalkaliphilic sulfate-reducing bacteria in soda lakes and the description of *Desulfonatronum thioautotrophicum* sp. nov., *Desulfonatronum thiosulfatophilum* sp. nov., *Desulfonatronovibrio thiodismutans* sp. nov., and *Desulfonatronovibrio magnus* sp. nov. *Extremophiles*, *15*(3), 391-401.

Sorokin, D. Y., Merkel, A. Y., Ziganshin, R. H., & Kublanov, I. V. (2025). Growth physiology, genomics, and proteomics of *Desulfurivibrio dismutans* sp. nov., an obligately chemolithoautotrophic, sulfur disproportionating and ammonifying haloalkaliphile from soda lakes. *Frontiers in Microbiology*, *16*, 1590477.

Thorup, C., Schramm, A., Findlay, A. J., Finster, K. W., & Schreiber, L. (2017). Disguised as a sulfate reducer: growth of the deltaproteobacterium *Desulfurivibrio alkaliphilus* by sulfide oxidation with nitrate. *MBio*, *8*(4), 10-1128.

Umezawa, K., Kojima, H., Kato, Y., & Fukui, M. (2021). *Dissulfurispira thermophila* gen. nov., sp. nov., a thermophilic chemolithoautotroph growing by sulfur disproportionation, and proposal of novel taxa in the phylum Nitrospirota to reclassify the genus *Thermodesulfovibrio*. *Systematic and applied microbiology*, *44*(2), 126184.

Wang, S., Jiang, L., Xie, S., Alain, K., Wang, Z., Wang, J., Liu, D. & Shao, Z. (2023). Disproportionation of inorganic sulfur compounds by mesophilic chemolithoautotrophic Campylobacterota. *Msystems*, *8*(1), e00954-22.

Warthmann, R., Vasconcelos, C., Sass, H., & McKenzie, J. A. (2005). *Desulfovibrio brasiliensis* sp. nov., a moderate halophilic sulfate-reducing bacterium from Lagoa Vermelha (Brazil) mediating dolomite formation. *Extremophiles*, *9*(3), 255-261.

Wu, X. T., Qiu, M., He, Y. Q., Wu, K., Zhao, J. Y., Wang, J., Ren, H.-J., Su, J.-Y. & Bao, P. (2025). Disproportionation of elemental sulfur by Exiguobacterium from marine sediment. *ISME communications*, *5*(1), ycaf168.

Zhilina, T. N., Zavarzin, G. A., Rainey, F. A., Pikuta, E. N., Osipov, G. A., & Kostrikina, N. A. (1997). *Desulfonatronovibrio hydrogenovorans* gen. nov., sp. nov., an alkaliphilic, sulfate-reducing bacterium. *International journal of systematic bacteriology*, *47*(1), 144-149.
